# Liquid Metal Nanotransformers for Drug‐Resistant Pan‐Cancer Therapy in Patient‐Derived Organoids

**DOI:** 10.1002/advs.202521041

**Published:** 2026-04-13

**Authors:** Xiaojie Yuan, Xuelin Wang, Zhongyao Chen, Shuo Wang, Weining Gao, Ziyi Chen, Runyang Li, Cheng Hao, Lei Zhang, Qiongqiong Zhang, Ke Zhang, Mei Yu, Qian Liu, Jingxuan Wang, Huiping Li, Simpkins Fiona, Jing Liu, Qiang Liu, Peng Liu, Yawei Hu

**Affiliations:** ^1^ Changping National Laboratory Beijing China; ^2^ Department of Chemical Engineering Tsinghua University Beijing China; ^3^ School of Engineering Medicine Beihang University Beijing China; ^4^ School of Biomedical Engineering Tsinghua University Beijing China; ^5^ Center of International Innovation For Technology and Science Shenzhen China; ^6^ Department of Pediatrics Faculty of Medicine The Chinese University of Hong Kong Hong Kong China; ^7^ Peking University International Hospital Beijing China; ^8^ School of Clinical Medicine Tsinghua University (Beijing Tsinghua Changgung Hospital) Beijing China; ^9^ Department of Obstetrics and Gynecology Beijing Tsinghua Changgung Hospital, School of Clinical Medicine, Tsinghua Medicine Tsinghua University Beijing China; ^10^ Department of Obstetrics and Gynecology, Peking Union Medical College Hospital, Chinese Academy of Medical Sciences & Peking Union Medical College National Clinical Research Center for Obstetric & Gynecologic Diseases Beijing China; ^11^ School of Basic Medical Sciences Tsinghua University Beijing China; ^12^ Department of Obstetrics and Gynecology University of Pennsylvania Philadelphia Pennsylvania USA; ^13^ State Key Laboratory of Cryogenic Science and Technology Technical Institute of Physics and Chemistry Chinese Academy of Sciences Beijing China

**Keywords:** cryotherapy, drug‐resistance, liquid metal nanotransformer, pan‐cancer, patient‐derived organoids

## Abstract

Drug resistance remains a major obstacle in cancer therapy, and direct mechanical disruption of cellular structures offers a promising strategy for overcoming it, highlighting the urgent clinical need for an effective and universal pan‐cancer treatment. Here, a cryo‐responsive liquid metal (LM) nanotransformer platform is developed. It undergoes dramatic deformation under freezing, physically disrupting lysosomal membranes and inducing potent cytotoxicity, particularly against drug‐resistant tumors, thus offering a broadly applicable pan‐cancer treatment. To enhance deformation efficiency of LM transformers, bismuth (Bi) is incorporated into gallium (Ga) to form biphasic Bi‐Ga alloy particles, which reduces Ga supercooling and promotes crystallization during freezing. The biosafety of LM particles is evaluated across multiple organoid models upon maximum contact. Based on 11 tumor organoid models including lung, colorectal, and ovarian cancers, the significant anti‐tumor effects of LM‐assisted cryotherapy are demonstrated, particularly Bi‐Ga combined with chemotherapy illustrating the strongest anti‐tumor efficacy. Transcriptomic analysis reveals LM anti‐tumor mechanism on multiple cancers with altering gene expression related to necroptosis, metabolic regulation, and cellular stress. In addition, tumors receiving LM cryo‐treatment exhibited hallmark features of immunogenic cell death. In immune‐competent drug‐resistant lung organoids, Bi‐Ga cryo‐treatment modulates tumor microenvironment and enhances immunogenicity. Collectively, this study establishes a universal LM nanotransformer‐enabled pan‐cancer strategy, offering a clinically viable and drug‐free approach for overcoming drug resistance.

## Graphical abstract

Pan‐cancer therapies are severely limited in drug‐resistance patients due to genetic mutations and other factors, resulting in poor therapeutic outcomes and constrained clinical benefit. Liquid metal nanotransformers, a new class of shape‐transformable nanomaterials capable of dramatic morphological changes, offer a promising physical strategy to directly puncture tumor cell for broad‐spectrum tumor eradication and effective circumvention of drug resistance.

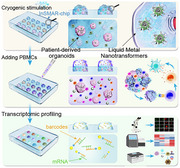

## 1 Introduction

One of the major challenges in cancer therapy is the emergence of drug resistance, mainly driven by factors including tumor heterogeneity, tumor microenvironment, cancer stem cells (CSC), and genetic mutations [1, 2]. These factors collectively hinder therapeutic efficacy and limit clinical outcomes. While personalized targeted therapies advance rapidly, their widespread clinical implementation remains restricted by high treatment costs and suboptimal effectiveness across diverse cancer types, leaving the majority of patients without viable treatment options [3, 4]. Nanotransformers, a class of nanomaterials capable of undergoing dramatic morphological changes to induce cell mechanical destruction, represent a powerful physical strategy to overcome core challenges of tumor drug resistance, including target‐dependent resistance, efflux pumps, apoptosis evasion, and CSC‐mediated resistance [1, 5, 6]. Unlike conventional drugs, their non‐biochemical mechanism disrupts cellular structures directly, enabling indiscriminate killing of even the most resistant tumor subpopulations, thereby realizing pan‐cancer eradication to circumvent conventional resistance mechanisms. Moreover, physical stimuli‐induced tumor killing elicits immunogenic cell death (ICD), which has been associated with enhanced intratumoral immune infiltration and improved therapeutic efficacy across multiple studies [7].Among candidate materials for constructing nanotransformers, low‐melting‐point metals (LMPMs), defined as metals and alloys with melting points below 300°C, have attracted increasing attention due to their characteristics for reversible liquid‐solid phase transitions [8, 9, 10, 11]. These transitions endow LMPMs with a shape‐transformable property highly suitable for responsive therapeutic applications [12]. Existing literatures have demonstrated that light‐, microwave‐ and freezing‐driven morphological transformations of liquid metal (LM) nanotransformers, from spherical to rod‐like structures, can physically disrupt cellular architecture, facilitating endosomal escape and drug release, thereby significantly enhancing anti‐tumor efficacy [8, 13, 14, 15]. Notably, gallium (Ga) and bismuth (Bi) based LMs, valued for their unique properties, including conductivity, functionalization, biocompatibility, and eco‐friendliness, have rapidly advanced diverse biomedical applications in tumor therapy, bone repair, bio‐imaging, and personal care [16, 17, 18]. Therefore, Ga‐ and Bi‐based LMs offer promising potential serving as nanotransformers to enhance pan‐cancer therapeutics, particularly against diverse drug‐resistance tumors. However, controlling such deformation and elucidating its pan‐cancer anti‐tumor mechanisms especially in drug resistance therapeutics remain key scientific challenges.

Meanwhile, to achieve clinically relevant validation of pan‐cancer therapies toward drug‐resistance models, we utilize here patient‐derived organoids (PDOs), the transformative in vitro model that preserve the genetic, phenotypic, and structural fidelity of primary tumors [19, 20]. PDOs faithfully recapitulate tumor complexity and therapeutic resistance, offering a powerful platform to evaluate emerging therapeutic strategies, particularly in cases of chemo‐resistant and immune‐evasive malignancies where conventional models fall short [21, 22]. However, conventional PDOs protocols typically require microliter‐scale cultures and extended in vitro expansion over several weeks to months to generate sufficient organoids for drug testing [23]. To overcome these limitations, we employed our previously developed high‐throughput, nanoliter‐scale integrated superhydrophobic microwell array chip (InSMAR‐chip) coupled with a mechanical tissue processing for establishment, three‐dimensional (3D) culture and high‐throughput profiling of PDOs without the need for passaging [24, 25, 26, 27]. Leveraging this robust and scalable platform, we aim to propose a clinically feasible LM nanotransformer strategy for pan‐cancer therapeutics, especially for tumors refractory to standard chemotherapy.

**FIGURE 1:**
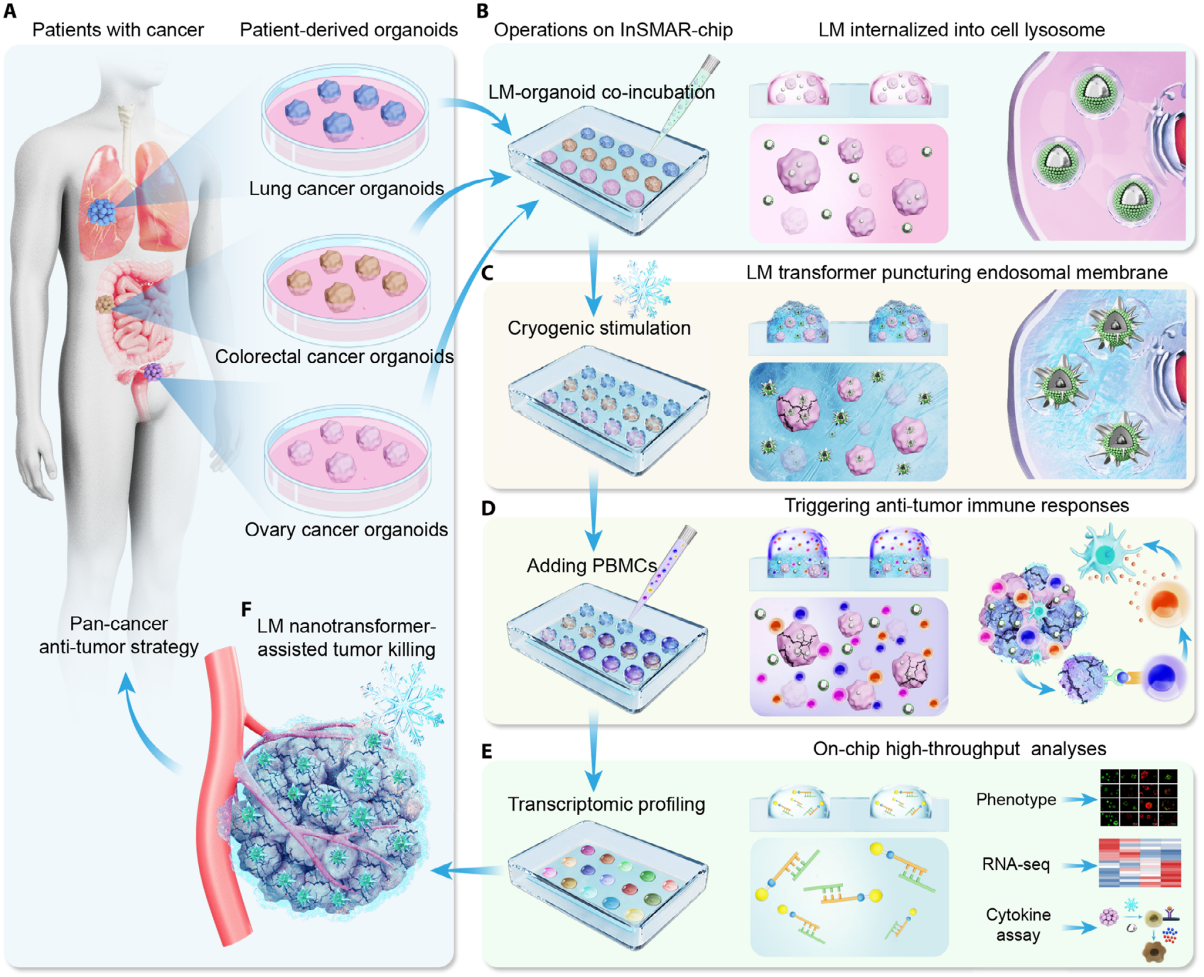
Schematic illustration of LM nanotransformer‐assisted pan‐cancer cryotherapy across PDOs. (A) Multiple primary tumor organoids from patients, including lung cancer (LC), colorectal cancer (CRC), and ovary cancer (OC). (B) LM particles were mixed with organoids and co‐incubated on the InSMAR‐chip, where they entered tumor cell lysosomes via endocytosis. (C) When applying cryogenic stimulation to the InSMAR‐chip, LM particles entered the organoids could transform into spike‐like structures, puncturing the endosomal membrane and enabling endosomal escape. (D) Organoid matched peripheral blood mononuclear cells (PBMCs) were added in the microwells after cryo‐treatment to assess immune activation. This setup demonstrated that LM‐facilitated cryoablation effectively triggered anti‐tumor immune responses. (E) Transcriptomic analyses were performed on treated organoids, revealing the potent tumor‐killing effect of Bi‐Ga and its ability to overcome drug resistance when combined with chemotherapy, as observed by phenotype. High‐throughput RNA‐seq further elucidated the underlying anti‐tumor mechanisms, while cytokine assays indicated enhanced antigen presentation and elevated levels of IFN‐γ and IL‐2. (F) The LM nanotransformer‐assisted pan‐cancer cryotherapy strategy could enable efficient tumor eradication in a drug‐free manner, overcoming the side effects of conventional chemotherapy and radiotherapy, and advancing a universal, non‐toxic, and broadly applicable cancer treatment approach.

In this study, we introduce cryogenic stimulation to trigger dramatic deformation of LM, enabling mechanical disruption of tumor cells for effective eradication of drug resistance pan‐cancers (Figure 1A). Cryoablation is a clinically applied, minimally invasive technique that eliminates tumors via localized freezing, offering advantages of real‐time imaging, reduced collateral damage, and immune activation through antigen release [28, 29, 30, 31]. To overcome its limitations in incomplete ablation and recurrence, we employ here Bi‐doped Ga (Bi‐Ga) LM as a synergistic medium to enhance tumor destruction and achieve more precise, reliable therapeutic outcomes. The addition of Bi will provide nucleation sites with Bi phase separation, and reduce the supercooling threshold of Ga with promoted crystallization, thereby establishing an unprecedented LM phase‐transition regulation strategy that enhances deformability under freezing. Across a range of drug‐sensitive and drug‐resistant PDO models including lung, colorectal, and ovarian cancers, Bi‐Ga nanotransformers are evaluated with highly efficient tumor infiltration, cellular uptake, and superior cryo‐induced cytotoxicity (Figure 1B,C). Furthermore, transcriptomic analyses reveals that Bi‐Ga‐mediated cryoablation can activate necroptosis, oxidative stress, and antigen presentation pathways, while suppressing proliferative and immunosuppressive signatures. In immune‐competent organoid models, we further demonstrate that Bi‐Ga cryoablation triggers strong cytokine responses to highlight its dual capacity for direct cytotoxicity and immune priming (Figure 1D,E). Overall, by integrating LM nanotechnology with PDOs and employing InSMAR‐chip, we present a universal and efficient strategy for pan‐cancer therapy, particularly targeting drug‐resistant tumors. This approach overcomes drug resistance through physical disruption while also activating immune‐related mechanisms, addressing the limitations of conventional physical ablation therapies (Figure 1F). It thus represents a new paradigm for pan‐cancer treatment, offering both precision and universality, while bridging gap between physical disruption and nanomedicine‐based therapy.

## 2 Results

### 2.1 Fabrication and Characterization of Bi‐Ga Transformer

**FIGURE 2:**
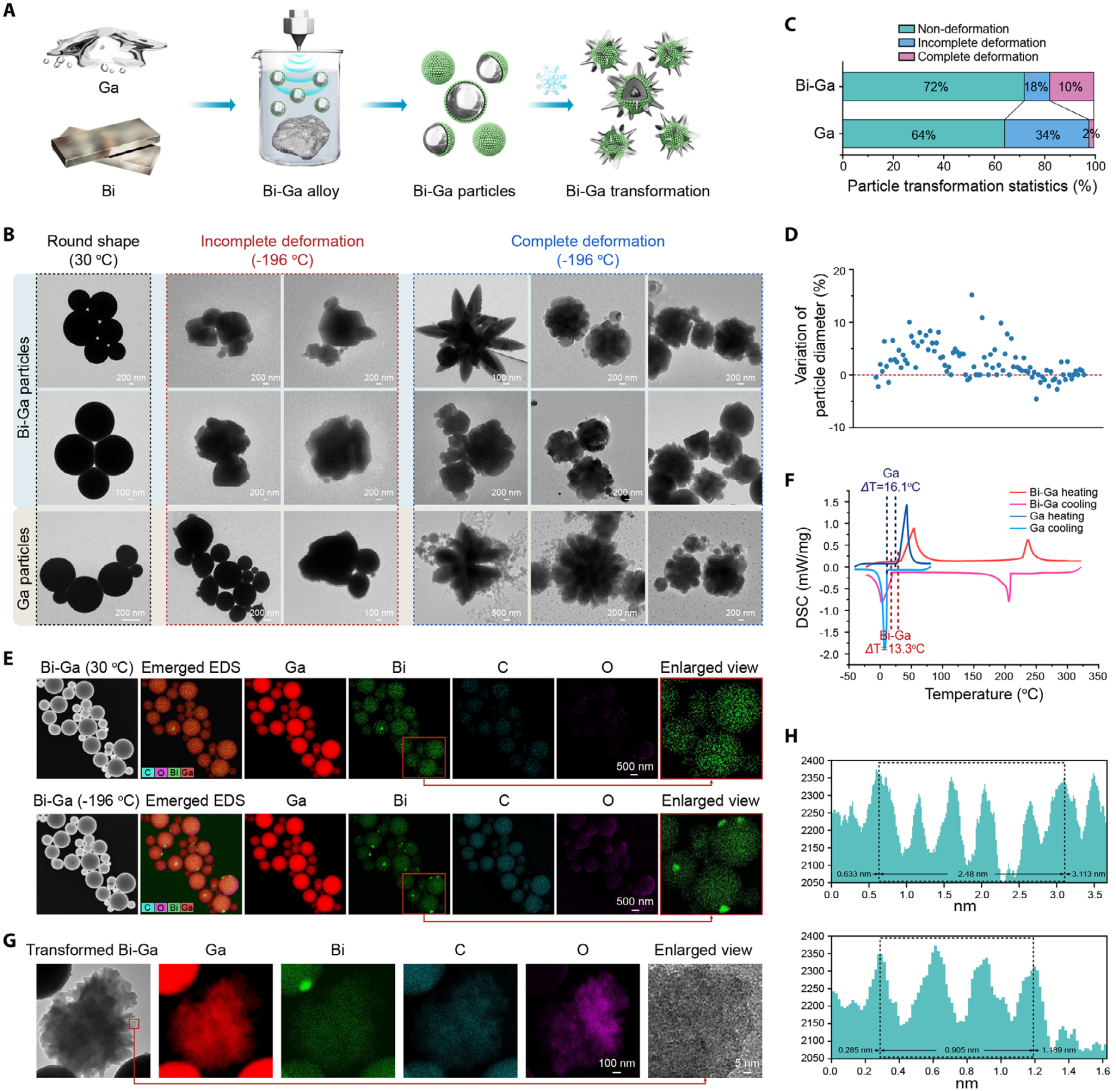
Fabrication and characterization of Bi‐Ga and pure Ga transformers. (A) Schematic illustration of the preparation and deformation processes of Bi‐Ga particles. (B) TEM and cryo‐TEM images of Bi‐Ga and Ga particles with round shape (30°C), incomplete deformation (−196°C) and complete deformation (−196°C). (C) Statistical analysis of Bi‐Ga and Ga particles deformation, with 200 particles analyzed for each type. (D) Variation of Bi‐Ga particles diameter, calculated as (diameter after freezing − diameter before freezing) / diameter before freezing, in total of over 100 particles, particle diameter was measured by in situ cryo‐TEM. (E) EDS results of Bi‐Ga particles at 30°C and −196°C, respectively, showing evident Bi phase separation under freezing, scale bar: 500 nm. (F) DSC measurements of Bi‐Ga and Ga during heating and cooling respectively, showing the decrease of Ga supercooling from 16.1°C to 13.3°C. (G) Transformed Bi‐Ga particle and corresponding EDS mapping showing the presence of Ga, Bi, C, and O within the transformer, along with an enlarged view of the transformer, scale bar: 100 nm. (H) Measured metal lattice spacing of the transformed Bi‐Ga particles with 0.496 and 0.302 nm.

While most metals exhibit positive thermal expansion during phase transitions, Ga‐ and Bi‐ based LMPMs are notable exceptions, displaying anomalous volumetric contraction upon melting, with volume changes of ‐3.05% and ‐3.08%, respectively [32, 33]. During freezing, Ga particles solidified from liquid and underwent dramatic morphological transformation into cactus‐like structures (Figure 2A,B) [8, 34]. However, micro/nano Ga particles, usually exhibiting extreme supercooling compared to their bulk counterparts (e.g., 35 nm Ga particles of 114.1°C supercooling and 3–15 nm Ga particles resisting freezing even below −183°C), show severely limited and uncontrollable deformation under cryogenic conditions, posing a major challenge to enhance the deformation efficiency for cryoablation augment [8, 35, 36]. To overcome this challenge, we further fabricated non‐eutectic biphasic Bi‐Ga alloy particles (termed Bi‐Ga particles) by doping Bi into Ga (Figure 2A). These particles appeared spherical under room temperature transmission electron microscope (TEM) but deformed under cryo‐TEM due to liquid‐solid phase transition (Figure 2B; Figure S1, Supporting Information). Cryo‐TEM revealed partially deformed Bi‐Ga particles exhibiting irregular morphologies between spherical and spiked ‘cactus‐like’ structures, defined here as incomplete deformation, while fully deformed cactus‐like structures were also observed with defining as complete deformation. Quantitative analysis of over 200 Bi‐Ga and Ga particles showed that pure Ga particles predominantly exhibited no or incomplete deformation under cryo‐triggering (Figure 2C). In contrast, Bi doping significantly increased deformation efficiency, with the proportion of completely deformed particles from 2% to approximately 10%. Particularly, cryo‐TEM imaging revealed that multiple simultaneously deformed particles were more frequently observed within a single field of view in Bi‐Ga samples, whereas such occurrences were rare in pure Ga samples (Figure 2B; Figure S2, Supporting Information). Meanwhile, with the assistance of in situ cryo‐TEM, we analyzed diameter changes in over 100 Bi‐Ga particles before (at room temperature) and after freezing (−196°C). Notably, more than 69% of the Bi‐Ga particles exhibited an increase in diameter upon freezing, consistent with the anomalous negative volume expansion of both Ga and Bi during the liquid‐solid phase transition (Figure 2D) [33].

Furthermore, elemental mapping by energy‐dispersive spectroscopy (EDS) at room temperature confirmed uniform Bi distribution within Ga, while the detected carbon (C) element originated from the polyethylene glycol (PEG) coating applied for particle stabilization and dispersion (Figure 2E). Upon cryo‐treatment, EDS revealed significant Bi segregation within Bi‐Ga particles, evidenced by bright, dispersed Bi element in the mapping. At the same time, differential scanning calorimetry (DSC) results confirmed that Bi precipitated first during cooling, followed by Ga‐based composite solidification (Figure 2F). Bi doping significantly reduced Ga supercooling from 16.1°C to 13.3°C, consistent with prior studies showing that metal doping lowers Ga supercooling, which promoted crystallization, and provided nucleation sites to enhance particle deformation efficiency [37, 38]. Besides, EDS analysis of deformed Bi‐Ga particles confirmed the presence of Ga, Bi, O, and C elements, verifying their constituent and excluding ice crystallization, thus strongly supporting their transformation in enhanced cryotherapy (Figure 2G; Figure S3A, Supporting Information). Further observation of high‐magnification imaging of deformed particle edges revealed metal lattice spacing of 0.496 nm and 0.302 nm (Figure 2H). The lattice spacing of the observed particle matched to the ‐201 (4.6790 Å) and 400 (2.9705 Å) crystal facets of β‐Ga_2_O_3_ (ICDD No.97‐002‐7699). Previous studies reported that the interfacial energy of β‐Ga is roughly one‐third that of α‐Ga, consistent with our finding that crystallization of liquid Ga at the micro/nanoscale preferentially formed β‐Ga rather than α‐Ga [39]. Besides, to evaluate batch‐to‐batch reproducibility, five independent batches of Ga particles and Bi‐Ga particles were prepared following the same fabrication procedure. The Ga particles showed an average hydrodynamic size of 1049.38 ± 60.37 nm with a PDI of 0.287 ± 0.028 (*n* = 5) and a zeta potential of −0.008 ± 0.031 mV, whereas the Bi‐Ga particles exhibited an average size of 967.5 ± 100.19 nm with a PDI of 0.323 ± 0.084 (n = 5) and a zeta potential of −0.024 ± 0.119 mV (Figure S3B,C, Supporting Information). Overall, this section demonstrated that Bi doping enhanced Ga nucleation and induced freezing expansion of Bi‐Ga particles, collectively boosting LM transformer deformation efficiency and offering a promising micro/nano medicine strategy for enhanced LM‐facilitated pan‐cancer cryotherapy.

### 2.2 Validations of Co‐Incubation and Biosafety of LM With Organoids

**FIGURE 3:**
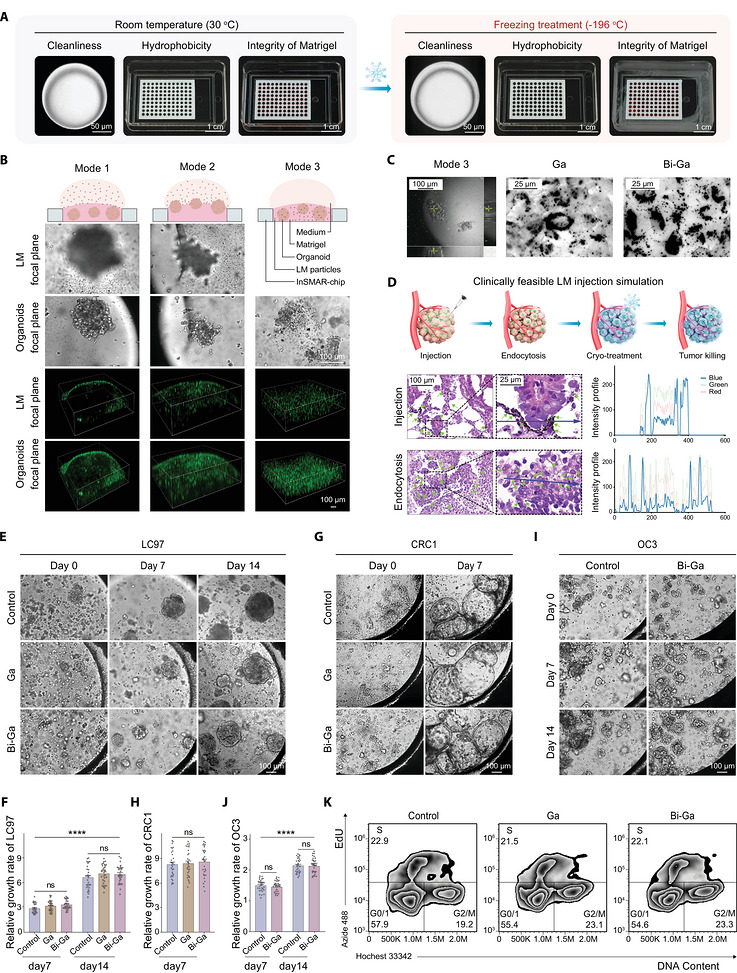
Optimization of LM particles inoculation modes and evaluation of biosafety across different tumor organoids. (A) Evaluation of InSMAR‐chip structural and functional integrity after cryo‐treatment. (B) Three inoculation modes between LM particles and organoids. LM particles here were modified with DSPE‐PEG FITC carrying green fluorescence (excitation wavelength: 494 nm, emission wavelength: 520 nm). The distribution of LM particles within the organoids was reflected by the respective focal planes and 3D reconstructed images under confocal microscopy. (C) The sufficient contact between LM particles and organoids under Mode 3 inoculation pattern, and the localization of LM particles within the cells after being internalized. The yellow cursor indicates the position of the LM particles. (D) Schematic illustration of clinically feasible LM particles injection and cryoablation process, and observations of LM particles injected and endocytosed into fresh clinical tumor tissues. H&E staining slices showed the uptake of LM particles by tumor tissue before and after 3 days of culture (green arrow: LM particles). In the intensity profile, the blue curve indicated the location of LM particles, while the red and green curves represented cytoplasm and other regions. (E, G, I) Images of three organoids co‐incubation with LM particles on InSMAR‐chip, showing the continuous growths of organoids from day 0 to 14. Scale bar: 100 µm. (F, H, J) The growth rates in three organoids after co‐incubation with Ga and Bi‐Ga particles for 7 and 14 days, N = 30 biologically independent organoids. Data are shown as mean ± SEM. Statistical significance was determined by one‐way ANOVA (****p < 0.0001; ns, not significant). (K) Cell cycle analysis of MC38 cells treated with Ga and Bi‐Ga compared with the control group by EdU, showing similar cell ratios in G0/1, G2/M, and S phase.

To assess the compatibility of the InSMAR‐chip with cryo‐treatment essential for LM particles activation, we evaluated its structural and functional integrity after treatment with liquid nitrogen (LN_2_) for 5, 10, or 20 min (Figure S4A). Microscopy revealed no observable contamination or structural damage within the microwells before and after freezing (Figure 3A). We further examined the surface hydrophobicity and the retention of Matrigel architecture, both of which remained unaffected after freezing (Figure 3A; Figure S4A, Supporting Information). Fluorescence imaging using serially diluted dyes showed signal intensities proportional to concentrations and strong inter‐row consistency across the chip (Figure S4B). These results collectively confirm the robustness and reliability of the InSMAR‐chip platform under cryogenic conditions.

To accurately evaluate the freezing‐induced cytotoxicity of LM particles on tumor organoids, it is essential to maximize their endocytic uptake by organoids. To this end, we labeled the LM particles with green fluorescence and investigated three distinct LM‐organoid co‐incubation modes using the InSMAR‐chip that we developed previously and visualized the 3D interactions via confocal microscopy (Figure 3B; Figure S5A,B, Supporting Information) [26]. Four hours after seeding, LM particles in Mode 1 and 2 remained distributed on the raised surface of the Matrigel. In contrast, in Mode 3, the LM particles were evenly embedded within the Matrigel (Figure 3B). After 3 days of incubation, organoids in Mode 1 and Mode 2 were in different focal plane, indicating limited or no contact. By contrast, in Mode 3, organoids and LM particles were located at the same focal plane, confirming direct interaction and spatial overlap (Figure 3B). To further evaluate LM particles uptake by organoids, 3D fluorescence projection imaging along the xz and yz planes revealed that LM particles were internalized into the organoids (Figure 3C; Figure S5C, Supporting Information). A similar pattern of uptake was observed in the MC38 cell line, confirming intracellular accumulation and endocytosis of LM particles (Figure 3C; Figure S6A, Supporting Information). Moreover, we utilized freshly resected patient tumor tissues to simulate the potential clinical scenario of intratumoral injection of LM particles (Figure 3D). Their distribution was subsequently assessed immediately after injection and at 3 days post‐injection using H&E staining. Initially, LM particles were predominantly retained at the tissue periphery without evident cellular uptake. After 3 days, they exhibited widespread distribution throughout the tissue, including clear intercellular localization, indicative of cellular internalization and diffusion (Figure 3D; Figure S7, Supporting Information). These findings support the potential of direct intratumoral injection of LM particles as a clinically applicable strategy for enhancing tumor penetration and efficient tumor killing.

We further evaluated the biosafety of Ga and Bi‐Ga particles in series of tumor organoid models, including lung cancer organoids lines (LC96, LC97), colorectal cancer organoid lines (CRC1, CRC2), and patient‐derived primary organoids from lung (LC1, LC2) and ovarian (OC1, OC2, OC3) cancers. Detailed information of all PDOs and organoid lines involved is in Tables S1 and S2 and their culture media recipe are in Table S3. In LC97, the organoid area increased approximately three‐fold by day 7 and six‐fold by day 14 (Figure 3E), with a statistically significant difference observed between day 7 and 14 (Figure 3F). Live/dead staining of organoid lines LC96 and LC97 after 3 days of co‐incubation with Ga and Bi‐Ga particles revealed no reduction in viability compared to control groups (Figures S8 and S9, Supporting Information), indicating no cytotoxic effects. Similarly, the CRC1 exhibited rapid expansion following LM particles exposure, reaching a diameter of approximately 300 µm and more than seven‐fold increase in area by day 7 (Figure 3G), with no impact on organoid growth rate (Figure 3H). Live/dead staining of CRC1 and CRC2 also confirmed the favorable biocompatibility of Ga and Bi‐Ga particles in colorectal organoids (Figures S10 and S11, Supporting Information). To validate biosafety for clinical samples, LM particles were also tested in primary PDOs. The OC3 organoid showed a 1.5‐fold increase in area by day 7 and more than a two‐fold increase by day 14 (Figure 3I), with a significant increase in growth between the two time points. Importantly, LM particles treatment did not impede organoid expansion (Figure 3J). Across all tested primary organoids (LC1, LC2, OC1, OC2, and OC3), no differences in cell viability were observed between LM‐treated and control groups (Figures S12 and S13, Supporting Information). We further investigated the effects of Ga and Bi‐Ga on the cell cycle using flow cytometry. The results showed that, compared with the control group, LM particles did not change the ratio of cells across different cell cycle stages, indicating no impact on DNA replication (Figure 3K). In summary, longitudinal growth tracking and viability analysis across multiple tumor organoids confirmed that LM particles had no adverse effect on organoid growth or viability, highlighting their excellent biosafety profile for potential therapeutic applications.

### 2.3 Freezing‐Triggered LM Transformer Promoting Tumor Organoids Killing

**FIGURE 4:**
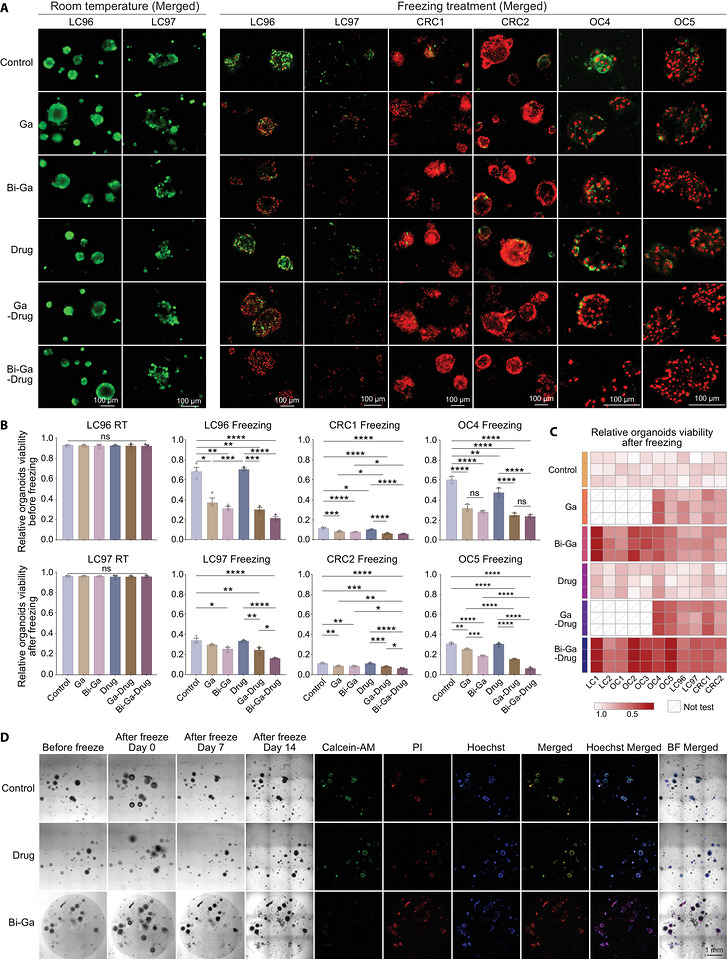
Evaluation of freezing‐triggered LM particles transformation in promoting tumor organoid killing, demonstrating the high efficacy and universality of LM‐enhanced cryotherapy for pan‐cancer treatment. (A) The live/dead staining of organoids with or without freezing after 3 days treatment of 6 groups (green: live cells; red: dead cells; blue: cell nucleus). Scale bar: 100 µm. (B) Relative viability of organoids with or without freezing after 3 days treatment of 6 groups. Data are shown as mean ± SEM. Statistical significance was determined by one‐way ANOVA (*p < 0.05, **p < 0.01, ***p < 0.001, ****p < 0.0001; ns, not significant). *n* = 3. (C) The heatmap represented the relative viability of 11 organoids after 3 days cryo‐treatment of 6 groups. The relative viability of each organoid sample was normalized to its own control group. Lower viability is indicated by a redder color. (D) Bright‐field stitched images of 3 groups (Control, Drug, Bi‐Ga) of LC96 at pre‐freezing, immediately post‐freezing, and on days 7 and 14 post‐freezing, along with live/dead staining fluorescence images on days 14 (green: live cells; red: dead cells; blue: cell nucleus). Scale bar: 1 mm.

To verify the freezing‐induced cytotoxicity of LM particles, we conducted live/dead staining on M38 cells and four tumor organoid lines (LC96, LC97, CRC1, and CRC2). Both Ga and Bi‐Ga groups exhibited significantly enhanced killing effects under freezing conditions compared to the non‐freezing counterparts and control groups (Figure 4A,B; Figures S6B,C and S8–S11, Supporting Information). To validate these findings across diverse tumor backgrounds, we extended the evaluation to multiple primary PDOs. In both LC1 and LC2 organoids, cell viability in the control groups remained above ∼80% after freezing, indicating intrinsic resistance to cryo‐induced damage. However, upon Bi‐Ga‐assisted cryotherapy, cell viability dramatically declined to ∼15% in LC1 and ∼50% in LC2, highlighting the enhanced tumoricidal effect of LM particles in cryo‐resistant tumors (Figure S12, Supporting Information). Similarly, in ovarian cancer organoids OC1, OC2, and OC3, cell viability remained high under room temperature conditions but decreased significantly following freezing treatment. Among them, even OC1 displaying relatively higher tolerance to cryo‐induced damage, the LM particles group showed excellent killing effect (Figure S13, Supporting Information). In OC4 and OC5, the combination of LM particles with cryo‐treatment further enhanced cytotoxicity compared to freezing alone (Figure 4A,B; Figures S14 and S15, Supporting Information). Collectively, these results suggest the broad applicability and robust tumor‐killing capacity of LM particles under freezing conditions across diverse organoid models.

Excitingly, across multiple organoid samples, LM particle‐assisted freezing showed superior cytotoxicity compared to chemotherapy alone, regardless of chemo‐sensitivity or chemo‐resistance. All chemotherapy drugs used here were standard first‐line clinical regimens for each cancer type: pemetrexed plus cisplatin for lung adenocarcinoma (LC96, LC1, and LC2), gemcitabine plus cisplatin for lung squamous carcinoma (LC97), irinotecan for colorectal cancer (CRC1 and CRC2), and paclitaxel plus carboplatin for ovarian cancer (OC4, OC5, OC1, OC2, and OC3). The information of chemotherapy drugs is listed in Table S4. Moreover, combining LM particles with chemotherapy could further enhance the tumor‐killing effect upon freezing. This synergistic effect between LM particles and chemotherapy under freezing conditions is likely attributable to the enhanced intracellular delivery of chemotherapy drugs facilitated by LM particles uptake, partially overcoming drug resistance. Across all organoid samples (including 7 primary PDOs and 4 organoid lines), LM particles treatment under freezing conditions yielded significantly greater cytotoxicity than control groups, with Bi‐Ga generally demonstrating higher efficacy than Ga in several organoids (Figure 4A–C; Figures S8–S15, Supporting Information).

To further evaluate the long‐term therapeutic efficacy of LM‐assisted cryoablation, we conducted a ‘resurgence’ experiment to monitor the regrowth and confirmed the final tumor‐killing effect of each group. Whole‐mount imaging of entire Matrigel droplets revealed that, prior to freezing, organoids exhibited intact and translucent. After freezing, these structures became disorganized and darker in appearance (Figure 4D). By day 14 post‐freezing, live/dead staining showed residual viable cells in the control and chemotherapy groups. In contrast, LM particle‐treated groups showed near‐complete loss of viability (Figure 4D), and the addition of chemotherapy further enhanced the cytotoxic effects, with Bi‐Ga showing superior efficacy to Ga (Figure S16, Supporting Information). In summary, these results provide compelling evidence that LM nanotransformers enhance pan‐cancer cryotherapy across a spectrum of tumor organoid models. This approach offers an effective and universal tumor treatment strategy for future clinical management of drug‐resistant malignancies.

### 2.4 Transcriptional Anti‐Tumor Mechanism of LM‐Assisted Cryotherapy on Multiple Cancer Organoids

**FIGURE 5:**
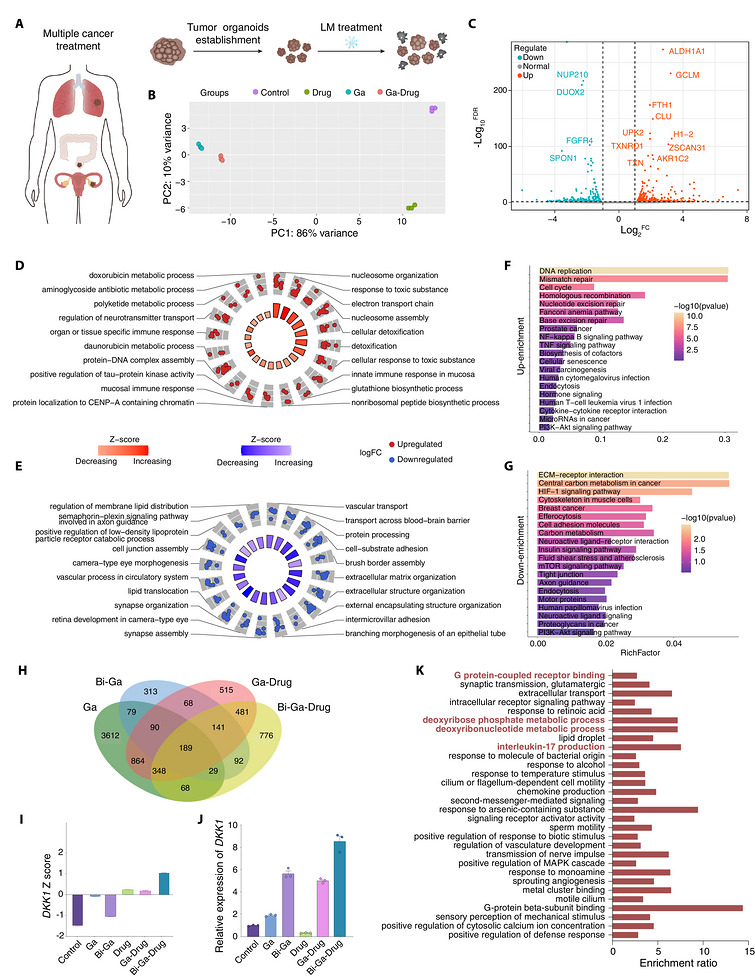
LM cryo‐treatment demonstrating anti‐tumor effects on multiple cancer organoids. (A) Schematic figure of LM‐assisted pan‐cancer cryoablation process. (B) Principal component analysis (PCA) plot of cryo‐treatment on CRC organoids. (C) Volcano plot of Ga cryo‐treatment on CRC organoids. (D) Gene ontology (GO) enrichment analysis of up‐enriched pathway by Ga cryo‐treatment on CRC organoids. (E) GO enrichment analysis of down‐enriched pathway by Ga cryo‐treatment on CRC organoids. (F) Kyoto Encyclopedia of Genes and Genomes (KEGG) enrichment analysis of up‐enriched pathway by Bi‐Ga cryo‐treatment on LC organoids. (G) KEGG enrichment analysis of down‐enriched pathway by Bi‐Ga cryo‐treatment on LC organoids. (H) Venn diagram of up‐regulated different expressed genes (DEGs) by LM cryo‐treatment on LC organoids. (I) Z‐score analysis of Bi‐Ga and Ga cryo‐treatment up‐regulated DEG DKK1 in LC organoids. (J) DKK1 mRNA expression levels in H23 cells after cryo‐treatment. Data are shown as mean ± SEM. Statistical significance was determined by one‐way ANOVA (*p < 0.05, **p < 0.01, ***p < 0.001, ****p < 0.0001; ns, not significant). *n* = 3. (K) Top 30 GO and KEGG up‐enriched pathway by LM cryo‐treatment on LC organoids.

To investigate the anti‐tumor effect of LM‐assisted cryo‐treatment on transcriptional level, we conducted Grouped‐seq on InSMAR‐chip for bulk RNA sequencing on different freezing treatment upon CRC and LC organoids (Figure 5A; and Table S5, Supporting Information) [26]. First, regarding to CRC organoids, PCA plot showed obvious different clusters among different treatments (Figure 5B). As shown in Figure 5C, Ga cryo‐treatment generated a variety of differentially expressed genes (DEGs) compared to control in CRC organoids. Pathways related to glutathione biosynthetic process, polyketide metabolic process and necroptosis were significantly up‐enriched (Figure 5D; Figure S17A, Supporting Information), while cell substrate adhesion, lipid translocation and extracellular matrix (ECM) receptor interaction related pathways were down‐enriched in Ga cryo‐treatment (Figure 5E; Figure S17B, Supporting Information), suggesting its potential metabolic regulation, necroptosis induction and barrier broken effect. Besides, oxidative stress and cryo‐treatment related genes were up‐regulated in both Ga and Ga‐Drug combined treatment compared to control in CRC organoids (Figure S17C, Supporting Information), indicating the effective anti‐tumor effect of LM cryo‐treatment upon CRC on transcriptional level.

Next, we applied Bi‐Ga cryo‐treatment and their combined treatment with anti‐tumor drug to LC organoid samples, PCA and volcano plot showed similar trend compared to CRC organoids (Figure S18A,B, Supporting Information). Bi‐Ga cryotherapy also demonstrated up‐enrichment pathways including double‐strand break repair, endocytosis and NF‐κB signaling pathway (Figure 5F; Figure S18C, Supporting Information). Tight junction, collagen‐containing ECM and growth factor binding related pathways were down‐enriched compared to control in LC organoids (Figure 5G; Figure S18D, Supporting Information), which showed similar treatment effect with CRC organoids. And the endocytosis up‐regulated mechanism was also consistent with our previous publication in which LM nanotransformers pierced the endosomal membrane under freezing, facilitating endosomal escape [8]. When comparing the effect of Bi‐Ga and Ga cryo‐treatment on LC organoids, we found that ionic homeostasis, cell proliferation related gene expression was lower in Bi‐Ga treatment, while stress and tumor metastasis related gene expression was higher in Bi‐Ga treatment compared to Ga treatment (Figure S18E,F, Supporting Information), suggesting the better performance of Bi‐Ga than Ga cryo‐treatment in terms of anti‐tumor effect.

Subsequently, to explore the potential of LM cryo‐treatment on multiple cancers, we conducted Venn diagram for CRC organoids, LC organoids and both CRC and LC organoids with all LM cryo‐treatment. As shown in Figure S19A and Table S6, Ga and Ga‐Drug treatment had well overlapped DEGs in both up‐and down‐regulated direction compared to control in CRC organoids. Consistent with the previous findings, overlapped up‐DEGs were enriched to stress response to LM ion and endocytosis‐related pathways (Figure S20A,B, Supporting Information), while ECM‐receptor interaction and hormone metabolic process were down‐enriched by down‐overlapped DEGs in CRC organoids (Figure S20C,D, Supporting Information). Regarding to LC organoids, a well‐overlapping relationship could also be found in both up‐and down‐DEGs compared to control (Figure 5H; Figure S19B and Table S7, Supporting Information). G protein‐coupled receptor binding and interleukin‐17 production related pathway were found up‐enriched in LC organoids using overlapped up‐DEGs in all LM cryo‐treatment (Figure 5K), while hormone metabolic process and gap junction‐related pathways were down‐enriched in all LM cryo‐treatment in LC organoids (Figure S20E).

For the Bi‐Ga unique DEGs in the Venn diagram (Figure 5H; Figure S19B, Supporting Information), neuropeptide receptor binding, MAPK and PI3K‐Akt signaling pathways were up‐enriched (Figure S21A,B, Supporting Information) and voltage‐gated channel activity and cell adhesion molecules related pathways were down‐enriched (Figure S21C,D, Supporting Information), suggesting the Bi‐Ga cryo‐treatment possessed advantages in apoptotic and channel activity regulation than other treatments. DKK1, a gene related to cell junction disassembly, was up‐regulated especially in LM and drug combined therapy (Figure 5I), the result was further validated in human lung cancer cell line H23 cells by qPCR (Figure 5J). Besides, FTH1 and SLC7A11, related to ferroptosis and metabolism, were also significantly up‐regulated with the validation by qPCR in H23 (Figure S25B,C, Supporting Information), respectively.

Then, to validate the overlap treatment effects of Ga cryoablation on both CRC and LC, overlapped Ga up‐and down‐DEGs were used for pathway enrichment analysis. We found that Ga could up‐enrich RNA splicing, ribosome and endocytosis pathways (Figure S21E,F, Supporting Information) and down‐enrich ATPase‐coupled transmembrane transporter activity and thyroid hormone signaling pathways (Figure S21G,H, Supporting Information), further confirmed the anti‐tumor effects of Ga cryo‐treatment on both CRC and LC organoids. As for all types of LM cryo‐treatment in all tumor organoids, although the up and down DEGs were not well overlapped (Figure S19C, Supporting Information), interleukin‐17 production and cell junction disassembly pathways were up‐enriched (Figure S20F, Supporting Information) and neutral lipid metabolic process pathway was down‐enriched (Figure S20G, Supporting Information) by applying overlapped DEGs in all cancer. All the above results suggested that LM‐assisted cryotherapy exerted potent anti‐tumor effect on multiple cancers.

### 2.5 Anti‐Tumor Effect of LM‐Assisted Cryotherapy on PDOs With Immune Microenvironment

**FIGURE 6:**
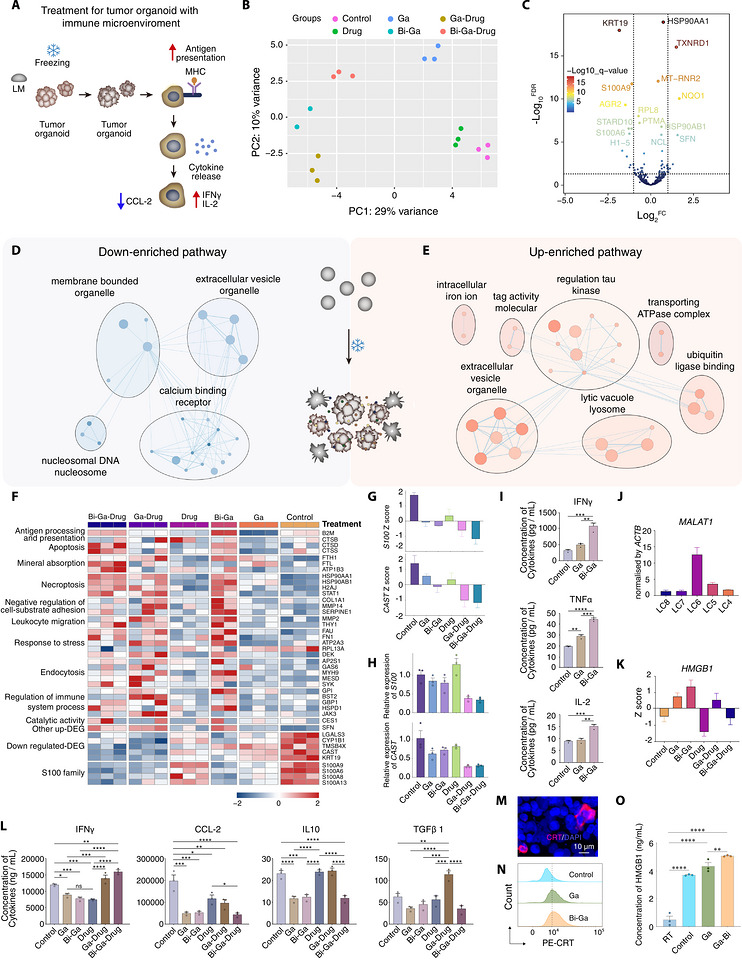
Anti‐tumor effect on LC organoids with immune compartment by LM cryo‐treatment and drug combined treatment. (A) Schematic illustration of LM cryo‐treatment for LC organoids with immune microenvironment. (B) PCA plot of different cryo‐treatments on LC organoids with immune microenvironment. (C) Volcano plot of Bi‐Ga‐Drug cryo‐treatment on LC organoids with immune microenvironment. (D) GO and KEGG down‐enriched pathway by Bi‐Ga‐Drug cryo‐treatment on LC organoids with immune microenvironment. (E) GO and KEGG up‐enriched pathway by Bi‐Ga‐Drug cryo‐treatment on LC organoids with immune microenvironment. (F) Heatmap of Bi‐Ga‐Drug cryo‐treatment on LC organoids with immune microenvironment. (G) Z‐score analysis of Bi‐Ga and Ga cryo‐treatment down‐regulated DEGs in LC organoids with microenvironment derived from clinical samples (CAST, S100). (H) mRNA expression levels of CAST and S100 in LC9 organoids co‐cultured with PBMCs derived from clinical samples after cryo‐treatment. (I) LEGENDplex assays of immune‐activating cytokine in the immune microenvironment of LC organoids after Bi‐Ga cryo‐treatment. (J) Drug resistant gene *MALAT1* relative expression on different LC organoids. (K) Z‐score analysis of selected DEGs by Bi‐Ga‐Drug cryo‐treatment on drug resistant LC organoids with immune microenvironment. (L) LEGENDplex assays of immune‐activating/suppressive cytokines in the immune microenvironment of LC organoids after Bi‐Ga cryo‐treatment. Data are shown as mean ± SEM. Statistical significance was determined by one‐way ANOVA. *n* = 3. (M) Representative image of Immunofluorescence staining showing CRT translocation on H23 cell surface by the confocal microscopy after freezing with LM particles. Scale bar 10 µm. (N) Flow cytometry analysis of CRT expression on the surface of H23 cells with or without cryo‐treatment within 4 h. (O) HMGB1 release from H23 cells in the supernatant 24 h after cryo‐treatment. *n* = 3. Data are shown as mean ± SEM. Statistical significance was determined by one‐way ANOVA (*p < 0.05, **p < 0.01, ***p < 0.001, ****p < 0.0001; ns, not significant). *n* = 3.

We then explore the efficiency of LM cryo‐treatment on PDOs with immune microenvironment (Figure 6A). First, LM cryo‐treatment without drug combination was applied to LC organoids with obvious different transcriptional clusters compared to control (Figure S22A, Supporting Information). The Bi‐Ga group also exhibited a larger number of DEGs (Figure S22B, Supporting Information) and greater separation in the PCA plot compared to the Ga group (Figure S22A, Supporting Information). Apart from apoptosis, necroptosis and endocytosis pathways related genes which have been shown in the previous results, leukocyte migration, regulation of immune system process and catalytic activity pathways related genes were up‐regulated (Figure S22C,D, Supporting Information) and epithelial‐to‐mesenchymal transition and S100 family genes (Figure S22E, Supporting Information) were down‐regulated after Bi‐Ga cryo‐treatment, suggesting the potential antigen presentation and immune regulation effects of cryo‐treatment for lung cancer microenvironment. Furthermore, Bi‐Ga cryo‐treatment could drive programmed cell death pathway up‐enriched and cell adhesion activation pathway down‐enriched (Figure S22F and Table S8, Supporting Information) compared to control, which also consistent with our previous finding in tumor organoids.

Subsequently, with the combination of drug therapy, we found that Bi‐Ga‐Drug cryo‐treatment demonstrated obvious different clusters with control group (Figure 6B; Figure S23A–C, Supporting Information) and showed various of DEGs as well (Figure 6C). Ubiquitin ligase binding and intracellular iron ion related pathways were up‐enriched and calcium binding receptor related pathway was down‐enriched after Bi‐Ga‐Drug cryo‐treatment on LC organoids (Figure 6D,E; and Table S9, Supporting Information). Additionally, with the combination of drug therapy, antigen processing and presentation and stress response related genes were up‐regulated in both Bi‐Ga and Bi‐Ga‐Drug group (Figure 6F), suggesting drug and LM cryo‐treatment had a synergistic effect in anti‐tumor effect. Stress response, necroptosis, ribosome damage related genes were up‐regulated (Figures S23D–F and S25B, Supporting Information) and S100 family, proliferation and tumor metastasis related genes were down‐regulated (Figure 6G; Figure S23D–F, Supporting Information) in Bi‐Ga‐Drug group after cryo‐treatment on multiple LC organoid samples, which were validated by qPCR (Figure 6H; Figure S25C), further suggesting the consistency of Bi‐Ga‐Drug cryo‐treatment effects on lung cancer organoids with different clinical background.

Finally, *MALAT1*, an anti‐tumor drug resistant gene, was found obvious higher expression in LC6 organoids than other samples (Figure 6J). To explore the efficiency of cryo‐treatment on drug resistant lung cancer samples, we applied different treatments on the LC6 organoids. PCA plot showed drug only treatment had relatively less obvious transcriptional pattern than other treatments (Figure S24A, Supporting Information). Although the heatmap did not show obvious difference upon necroptosis, endocytosis, response to stress and regulation of immune system process related gene expression on different treatments (Figure S24B, Supporting Information), DNA damage, cryoablation induced cellular stress and antigen presentation related genes were up‐regulated in Bi‐Ga or Ga cryo‐treatment than with drug combination (Figure 6K; Figure S24C, Supporting Information). Furthermore, we found that Bi‐Ga cryo‐treatment could significantly enhance the secretion of immune‐activating cytokines and reduce immunosuppression compared to the Ga cryo‐treatment and combination group (Figure 6I,L; Figure S25A, Supporting Information). The above data indicated Bi‐Ga cryoablation showed more benefits to drug resistant cancer treatment than drug related treatment.

Further, apoptosis, necroptosis, and antigen processing and presentation pathway‐related genes as well as *HMGB1*, were up‐regulated in Ga and Bi‐Ga‐containing groups, implicating LM in the induction of ICD. H23 cells were co‐incubated with LM for 24 h, followed by snap‐freezing in LN_2_ for 10 s. CRT translocated to cell surface was assessed by immunofluorescence and flow cytometry within 4 h. Immunofluorescence staining revealed prominent CRT translocation to the H23 cell surface in the Ga and Bi‐Ga groups compared with the control group (Figure 6M; Figure S26, Supporting Information). Flow cytometry analysis further demonstrated increased CRT expression on the surface of H23 cells in the LM‐treated groups(Figure 6N). Secreted HMGB1, the late‐phase ICD marker was subsequently assessed by Elisa. In H23 cells, cryo‐treatment enhanced HMGB1 expression, and this effect was further potentiated when combined with LM particles (Figure 6O). We subsequently validated these findings in LC10 organoids derived from clinical tissue and pleural effusion specimens. The results demonstrated that cryo‐treatment combined with Bi‐Ga significantly increased HMGB1 release compared with cryotherapy alone (Figure S25E, Supporting Information).

In summary, LM‐assisted cryotherapy showed significant anti‐tumor effects on multiple cancers, cancers with immune microenvironment and drug‐resistant cancer, providing a novel feasible approach for clinical pan‐cancer treatment and personalized treatment strategy.

## 3 Discussion and Conclusion

Facing the drug‐resistance challenge, we report here a LM nanotransformer strategy to enhance high‐efficient pan‐cancer cryoablation. Our findings demonstrate that these cryo‐activated LM transformers exert potent mechanical disruption, induce ICD, and synergize with chemotherapy to overcome drug resistance in multiple PDO models, including lung, colorectal, and ovarian cancers. Our study presents several key innovations: (1) From the perspective of LM material science, we provide a deeper and more precise understanding of the deformation‐regulation mechanism of LMs, and for the first time, apply LM to clinical tumor samples, laying a foundation for future preclinical anti‐tumor validation. (2) In addressing drug resistance, we establish a universal and straightforward strategy based on LM nanotransformer‐induced mechanical disruption to efficiently kill a wide range of solid tumors, offering strong clinical translational potential. (3) From the organoid perspective, the high‐throughput organoid‐on‐chip platform used here enables cost‐effective and efficient evaluation of diverse clinical samples, supports tumor microenvironment reconstruction and molecular mechanism exploration, and offers high clinical relevance. Followings are the detail discussion.

First, we established a fundamentally new LM phase‐transition‐regulated nanotransformer strategy driven by freezing stimulation. By directly comparing pure Ga and Bi‐Ga alloy systems, we demonstrated that their distinct deformation behaviors translated into differential cytotoxic outcomes, with Bi‐Ga more effectively overcoming drug resistance. Mechanistically, Bi incorporation serves as heterogeneous nucleation sites, lowering the nucleation barrier and undercooling threshold of Ga, thereby accelerating crystallization and markedly enhancing particle deformability under cryogenic conditions. As we know, low temperatures can induce phase transitions and crystallization of fluids [40, 41] and similarly serve as a trigger for liquid‐solid phase transition in LM transformer. Exploring these unconventional phase transition dynamics will provide deeper insights into the fundamental properties and regulation of LM systems. The regulation mechanisms involved here, promote LM heterogeneous nucleation and reduced undercooling, enable efficient freezing‐induced deformation in Bi‐Ga particles, distinct from previously reported light‐ or microwave‐driven LM transformations that rely on thermal expansion or shape change for endosomal escape or drug release [13, 14, 15]. The dramatic phase transition‐induced deformation involves complex, synergistic phase transition mechanisms between metallic and non‐metallic fluids, coupled with pronounced size‐dependent effects [35, 36]. Collectively, these findings hold significant theoretical implications for the physics of LM phase transitions, micro/nanoscale heat transfer, cryoablation medicine, and materials science. By leveraging cryo‐triggered mechanical disruption, our platform circumvents reliance on biological pathways that are often dysregulated in drug resistant tumors [42], offering a physical mechanism of action that is broadly applicable across multiple cancers.

Second, minimally invasive treatments such as microwave, radiofrequency, and cryoablation show promising clinical outcomes but still face high postoperative recurrence rates, limiting curative potential [43]. Here, we propose a novel strategy, integrating cryoablation with functional mediators such as LM, to achieve more reliable tumor destruction and meet the demands of precise cancer therapy. In our study, we address the clinical cryoablation limitation of incomplete tumor eradication and recurrence by engineering Bi‐Ga nanotransformers to amplify cryoablation efficacy [44]. Upon freezing, the spiked morphologies mechanically disrupt tumors, while activation of stress pathways (e.g., oxidative stress, DNA damage) further augments cytotoxicity. This dual mode of action explains the superior performance of Bi‐Ga over Ga alone, as Bi doping enhances deformation efficiency and amplifies biological effects. Moreover, the synergistic effect of Bi‐Ga nanotransformers with chemotherapy underscores a promising combinatorial treatment strategy. By facilitating intracellular drug delivery through mechanical membrane disruption, Bi‐Ga nanotransformers overcome barriers to drug uptake in resistant tumors. This is particularly evident in chemo‐resistant PDOs, where the combination of Bi‐Ga cryotherapy and standard regimens (e.g., pemetrexed/cisplatin for lung cancer, paclitaxel/carboplatin for ovarian cancer) produce greater cytotoxicity compared to either treatment alone. Such synergy may reduce the required drug doses, potentially minimizing off‐target toxicity. Freezing serves as a clinically approved cryoablation strategy, and the mechanical disruption caused by spiked Bi‐Ga structures bypasses resistance mechanisms that limit heat‐driven or drug‐based approaches. Overall, our findings suggest a more effective and precise tumor‐killing approach than conventional cryotherapy. Future clinical trials will further evaluate its potential to improve cure rates by fully eradicating tumors and inducing immune activation, potentially reducing recurrence rates to levels comparable to or even lower than surgical resection, finally offering a minimally invasive therapy that may significantly extend survival for patients with malignant tumors.

Third, the use of PDOs and InSMAR‐chip enhances the translational relevance of our findings [24, 26, 27]. PDOs preserve the genetic and phenotypic heterogeneity of primary tumors, including drug resistant features, making them superior to cell lines for evaluating therapeutic responses [45]. The InSMAR‐chip exhibits structural integrity under cryogenic conditions, enabling high‐throughput screening of Bi‐Ga efficacy across multiple PDOs. This platform bridges the gap between preclinical research and clinical application, allowing for rapid assessment of treatment responses in a patient‐specific manner. In addition, this marks the first application of LM in clinical tumor samples, representing a small but meaningful breakthrough. It lays the groundwork for future clinical translation of LM and offers a more effective, physiologically relevant validation approach that better reflects real‐world therapeutic responses. In our study, we fully elucidate the underlying LM anti‐tumor mechanisms by transcriptomic analyses. Bi‐Ga‐assisted cryoablation activates necroptosis, oxidative stress, and antigen presentation pathways associated with ICD, while suppressing proliferative and immunosuppressive signatures. In immune‐competent PDO models, this translates into enhanced secretion of immune‐activating cytokines and reduces immunosuppression, consistent with the notion that cryoablation can stimulate anti‐tumor immunity. Notably, these effects are amplified in drug‐resistant PDOs, where Bi‐Ga cryotherapy outperforms standard chemotherapy regimens, highlighting its potential to bypass resistance mechanisms driven by genetic or epigenetic alterations [46]. Therefore, based on our pan‐cancer PDOs and InSMAR‐chip platform, we enable efficient preclinical ex vivo antitumor screening. For patients unresponsive to chemotherapy, this strategy offers a simplified alternative to complex genomic profiling and targeted drug selection, allowing effective tumor eradication via LM transformers while avoiding resistance caused by mutation‐driven drug escape.

Despite promising results, several limitations warrant consideration. First, this work did not involve in vivo experiments; therefore, the biodistribution and targeting efficiency of the particles following systemic administration were not evaluated. Without specific targeting strategies, intravenously delivered nanoparticles may not achieve efficient accumulation at tumor sites. Future studies may address this limitation by introducing targeting modifications, such as homologous tumor cell membrane coating as reported in our previous work [8]. Second, cryo‐treatment of LM, although effective in PDOs, may limit applicability to inaccessible or metastatic tumors. Targeting modification combined with systemic delivery may enable accumulation in deep tumor regions, but the targeting efficiency and potential limitations associated with diffuse metastatic lesions warrant further investigation. Third, the mechanisms underlying Bi segregation during freezing and its role in immune activation remain to be fully investigated for further optimization. Forth, integrating Bi‐Ga cryotherapy with immunotherapies (e.g., immune checkpoint inhibitors) can further enhance anti‐tumor immunity, as cryo‐induced antigen release and reduced immunosuppression may promote T cell infiltration [47, 48, 49, 50]. Moreover, although previous studies suggest that Ga‐based particles may undergo partial degradation in vivo with the release of Ga^3^^+^ ions and accumulation in organs such as the liver [51, 52], their detailed metabolic pathways and long‐term biosafety require further systematic investigation. In addition, while our earlier work indicated that Ga particles can be largely metabolized within approximately 28 days [8], comprehensive in vivo evaluation will still be necessary for clinical translation. Besides, exploring Bi‐Ga X‐ray visibility will enable image‐guided cryoablation, improving precision in clinical applicability. Finally, drug resistance arises not only with chemotherapy but also with small‐molecule targeted therapies and antibody‐based drugs due to genetic mutations or other reasons. [4, 53] In the future, we will collect a broader range of clinical samples resistant to various frontline treatments, including targeted and antibody therapies, to expand testing across diverse drug‐resistant tumor types and further validate the pan‐cancer potential of this strategy. In summary, Bi‐Ga LM nanotransformers represent a paradigm shift in pan‐cancer therapy, combining physical disruption with immune activation to overcome drug resistance. By utilizing cryo‐triggered morphological transformations and leveraging PDOs for preclinical validation, this approach bridges nanomedicine and cryoablation, offering a universal, cost‐effective strategy for diverse tumors. LM‐assisted cryoablation can also serve as a strategy to regulate the tumor microenvironment, potentially converting it from a “cold” to a “hot” state. In the future, it may be combined with immunotherapy to enhance cancer treatment. With continued development, LM‐assisted cryotherapy could redefine treatment standards for drug resistant cancers, providing an alternative to conventional therapeutics.

## 4 Methods

### 4.1 Experimental Design

The aim of this study is to explore the potential of LM particles in clinical tumor therapy by using the in vitro model of patient‐derived primary tumor organoids from clinical tissues or organoid lines and InSMAR‐chip. For the generation of patient‐derived organoids, we used PDO lines either previously described [26, 27], and newly generated from patients with LC, OC, upon acquiring their written consent approved by the ethics review board of the Peking University International Hospital (Ethical Approval No.: 2025‐KY‐0051‐01), the Institutional Review Board (IRB) of Peking Union Medical College Hospital (PUMCH) (Ethical Approval No.: I‐25PJ1487); Ethics Committee of Beijing Tsinghua Changgung Hospital (Ethical Approval No.: 24575‐4‐01) approved the use of patients' discarded tissues and obtained primary organoids for the research of new tumor therapies. Pathology, TNM stage, gene mutation site, treatment in clinic, RECIST evaluation and other detailed information of all clinical samples are in Table S1. All regents and critical kit involved are in Table S10.

### 4.2 Preparation of Ga and Bi‐Ga Particles

Gallium (Ga, purity ≥ 99.99%) and bismuth (Bi, purity ≥ 99.99%) were purchased from Jinyuan Industrial Co. Ltd (China). Non‐eutectic biphasic Bi‐Ga alloy was prepared by melting 5.0 wt.% Bi and 95.0 wt.% Ga in a heating container at 300°C for 1 h. To prepare Ga and Bi‐Ga particles, 20 mg DSPE‐PEG2000 (Tanshtech, China) was dissolved in 10 mL deionized water, followed by adding 50 mg Ga or Bi‐Ga alloy. The mixture was sonicated by ultrasonic disruption system (Branson, USA) at 60% amplitude using a pulsed cycle (4 s ON, 4 s OFF) for 10 min under ice cooling. The prepared solution was then centrifuged at 800 rpm for 5 min to remove large aggregates, followed by a second centrifugation at 3000 rpm for 5 min to collect the particle pellets. Purified particles were resuspended in deionized water and stored at 4°C.

### 4.3 Characterization of Ga and Bi‐Ga Particles

Particle morphology was characterized using TEM (Hitachi 7650B, Japan) at ambient temperature. Cryo‐TEM (FEI Tecnai F20, Netherlands) was performed under LN_2_ conditions to assess morphology changes during freezing. Elemental mapping in Bi‐Ga particles was analyzed at −180°C using high‐resolution TEM (HRTEM, JEM‐2100Plus, Japan) equipped with energy‐dispersive X‐ray spectroscopy (EDS), acquiring elemental maps for Ga, Bi, O, and C to verify Bi phase separation under freezing. The same instrument facilitated in situ comparative diameter measurements before and after freezing. Thermal behavior was evaluated by differential scanning calorimetry (DSC, NETZSCH DSC 200F3Maia, Germany). Ga metal and Bi‐Ga alloy samples were scanned between −50°C and 350°C at heating/cooling rates of 10 K/min.

### 4.4 Fabrication and Performance Evaluation of InSMAR‐Chip

The InSMAR‐chip made of polycarbonate used in the experiment was injection molded after opening the mold [25]. To prepare the superhydrophobic coating, 0.25 g of 1H, 1H, 2H, 2H‐perfluorooctyltriethoxysilane (Aladdin) was added to 20 g of ethanol absolute (Sangon Biotech) and mixed thoroughly for 30 min. Then, 1.5 g Titanium Dioxide (TiO2) P25 (Degussa) and 1.5 g titanium oxide (Aladdin) were weighed, and the mixed liquid was added to the powder and shaken overnight. Then the superhydrophobic coating was drop onto the surface of the InSMAR‐chip and allowed to dry.

### 4.5 Generation of Primary Tumor Organoids Derived from Clinical Samples

The fresh tumor samples obtained from patients were preserved in preservation solution (DMEM/F12 containing 1% penicillin‐streptomycin (Gibco)) and processed within 1 day. Upon receipt, the samples were imaged first, then divided randomly into several pieces. One piece was fixed in 4% polyformaldehyde (Sigma‐Aldrich) for H&E (Hematoxylin and Eosin) staining. The remaining pieces are chopped finely using surgical scissors and resuspended in organoid basal medium (Advanced DMEM/F12 containing 1% penicillin‐streptomycin, 1% HEPES, and 1% Glutamax‐I). The tissues in suspension were transferred onto a 100 µm mesh filter (Falcon) and ground using a 5 mL syringe plunger. After rinsing the filter, residual impurities on the membrane were discarded along with the filter. Subsequently, the filtrate containing cell clusters and single cells was filtered through a 40 µm filter (Falcon) to collect cell clusters sized between 40 and 100 µm. The filter membrane was cut and placed into 2 mL complete factor medium and was washed to release the cell clusters into the medium. Then, the membrane was discarded, and the cell clusters were suspended and cultured overnight.

### 4.6 Culture, Passaging, and Cryopreservation of Cancer Organoids

Centrifuge the suspension containing organoids at 800 g for 5 min and discard the supernatant. Add an appropriate volume of Matrigel (Corning) and mix it thoroughly, then inoculate the mixed suspension into flat‐bottom multi‐well plates (Corning). Make the Matrigel to solidify for 30 min at 37°C, then add complete medium. The recipes of LC, OC and CRC organoid culture media are show in Table S3. Transfer the plates to the cell culture incubator at 37°C and 5% CO_2_. Replace the medium at least once a week. When culturing organoids on InSMAR‐chip, add 1 µL of Matrigel containing 5–10 organoids to each microwell. Add 1.5 µL complete medium on top of the Matrigel in each microwell. Due to the superhydrophobic properties of the InSMAR‐chip, the medium in each microwell will be independent. For the passaging of tumor organoids, 8 times the volume of organoid harvesting solution (R&D Systems) was added first, then placed on ice, and digested on a shaker for approximately 1 h to dissolve the Matrigel. By pipetting several times, the organoids are sheared into small clusters. Wash them with Advanced DMEM/F12 and resuspend them in Matrigel at a 1:2‐1:4 ratio for seeding. For cryopreservation, the culture medium was first removed by centrifugation, followed by resuspension in cold cryopreservation medium (CELLBANKER 2, Thermo Fisher). The suspension was then stored in a ‐80°C freezer overnight before transfer to LN_2_ for long‐term storage.

### 4.7 Optimization of Co‐Incubation Modes for LM Particles and Tumor Organoids

To improve the contact efficiency between organoids and LM, we explored 3 kinds of mixed inoculation modes. Mode 1: organoids were mixed in Matrigel for normal inoculation, and culture medium mixed with LM was added dropwise. Mode 2: organoids suspended in Matrigel were inoculated, and inverted on ice to allow the organoids to settle on the gel surface, then culture medium mixed with LM was added. Mode 3: organoids and LM were mixed evenly in Matrigel and then inoculated, followed by adding culture medium. All the above were inoculated on the InSMAR‐chip. Laser confocal microscopy (Nikon AXR NSPARC) was used to image the co‐existence of LM particles and organoids under the three co‐incubation models, respectively, to optimize the inoculation mode.

### 4.8 Organoid Cryo‐Treatment and Viability Assessment

Organoids were added with LM particles or chemotherapeutic drugs, and inoculated on InSMAR‐chip for 1 to 3 days. For operational convenience and experimental reproducibility, we adopted a simplified freezing protocol by directly placing the InSMAR chip on LN_2_ gently. In the pan‐cancer organoid killing experiments investigating the combination of LM and cryotherapy, the seeded InSMAR chips were subjected to a single freezing cycle on the LN_2_ surface for 6 min and then returned to room temperature for following treatment. For mechanistic exploration like RNA sequencing, chips were frozen for merely 10 s prior to RNA extraction, as prolonged freezing would result in organoid death and compromise the integrity of RNA retrieval. For viability assessment, ten‐fold diluted alamarBlue Cell Viability Reagent (Invitrogen) was added to the microwells of the organoids before and after freezing and incubated for 2 h. The absorbance at 590 nm was detected using an adapted microplate reader, and the ratio of background‐removed absorbance value after freezing to before freezing was the relative viability. To phenotypically assess the viability of organoids, we washed the organoids with PBS and stained them using the Calcein‐AM/PI Double Stain Kit (YEASEN) and Hoechst 33342 or DAPI (10 µg/ml, Invitrogen). The dyes were diluted 1000‐fold with PBS, added to the organoids, incubated at 37°C for 30 min. After washing off the remaining dyes, photographed using a confocal microscope (Nikon AXR NSPARC). Data were analyzed using Imaris 9.9. Relative organoid viability was calculated as the ratio of live cell number to total cell number, whereas the killing effect was defined as the ratio of dead cell number to total cell number. Total cell number was determined by counting all Hoechst‐stained nuclei; live cell number was quantified as nuclei within Calcein‐AM‐positive regions; and dead cell number was quantified as nuclei within PI‐positive regions.

### 4.9 H&E Staining of Cancer Tissue

The freshly resected cancer tissues were transported to the laboratory and divided into unbiased several pieces within a day. 40 µL of fibrinogen solution (10 mg/ml, Sigma‐Aldrich) was mixed with 20 µL of thrombin reagent (1000 U/mL, Solarbio) for fibrin polymerization to fixed samples to a 96‐well round‐bottom plate. U40 Insulin Syringe (BRAUN) was used to draw 200 µL of LM solution to inject into the ovarian cancer tissue. Half of the tissues were randomly selected and fixed with 4% paraformaldehyde, and the remaining tissues were cultured in suspension with organoid complete medium for three days, followed by fixation with 4% paraformaldehyde. The tissues were paraffin‐embedded and sectioned, and HE staining was performed to observe the distribution of LM in the tissues. After washing off the fixative, place the tissue in different concentrations of alcohol (70%, 80%, 90%, 95%, and 100%) sequentially, soaking for 1–2 h at each stage to gradually dehydrate the tissue. Then, place the tissue in xylene, soaking for 1–2 h (repeat 1–2 times) to make the tissue transparent. Immerse the tissue in melted paraffin, soaking for 1–2 h (repeat 2–3 times) to ensure sufficient paraffin penetration. Fix the paraffin block on the microtome, adjust the section thickness to 4–6 micrometers, and adhere it to poly‐L‐lysine‐coated slides. Place the slides in a 60°C oven for 30 min to ensure strong adhesion. Before staining, soak the slides in xylene for 3–5 min (repeat 2–3 times) to remove paraffin, then rehydrate through graded alcohol. Stain the slides with hematoxylin solution for 3–8 min, and rinse excess stain with tap water. Soak them in 0.5% HCl for 1–2 s, and immediately rinse the sections with tap water for 1–2 min to completely remove acidic substances. Stain them with eosin solution for 1–5 min, then rinse with tap water to remove excess stain. For transparency, soak the sections in graded alcohol for 1–2 min, then place them in xylene for 2–3 min (repeat 1–2 times). Add neutral mounting glue to the slides, cover with a coverslip, gently press to remove air bubbles, and let it dry at room temperature. Acquire images using the 3DHISTECH Panoramic SCAN system.

### 4.10 PBMCs Preparation

Add an equal volume of FACS buffer (PBS + 2% FBS + 1% P/S) to the blood and mix thoroughly. Add an equal volume of lymphoprep (STEMCEL) in an empty centrifuge tube and carefully layer the blood diluent on top of the lymphoprep. Centrifuge at 800 g with a ramp‐up/down of 4 for 30 min. Carefully aspirate the cloudy narrow band at the interface between the layers. Wash the cells twice with at least 5 volumes of FACS buffer. Lyse red blood cells using RBC lysis buffer (eBioscience). The isolated PBMCs are cultured in ultra‐low attachment plates (Coring) with T cell expansion medium (STEMCELL).

### 4.11 Cytokine Analysis with LEGENDplex

We seeded patient‐derived lung cancer organoids into microwells of the InSMAR‐chip, followed by adding PBMCs from the same patient after LN_2_ freezing. After co‐culture for 3 days, 6 groups of supernatants were collected separately, including control group, Ga co‐incubation group, Bi‐Ga co‐incubation group, chemotherapy drug group, Ga combined with chemotherapy drug co‐incubation group, and Bi‐Ga combined with chemotherapy drug co‐incubation group. The cytokine content in the culture supernatant was detected using LEGENDplex Multi‐Analyte Flow Assay Kit (Biolegend). The kit was pre‐warmed in room temperature for 30 min. Dilute standards. Take 250 µL of Assay Buffer to dissolve the Standard and mix well, let stand for 10 min, then transfer to EP tubes and label as C7. Then take 7 EP tubes, labeled as C6/C5/C4/C3/C2/C1/C0 respectively, add 75 µL of Assay Buffer to each tube, take 25 µL from C7 and perform serial four‐fold dilutions until C1, C0 is Assay Buffer (0 pg/µL). Dilute cell culture supernatant with Assay buffer. Add 25 µL of Assay buffer, 25 µL of Standard or sample, 25 µL of vortexed Premixed Beads to the 96‐well V‐bottom plate, with a total volume of 75 µL. Seal the plate with Plate Sealers, and cover it with aluminum foil to protect from light. Incubate at room temperature on a shaker at 800 rpm for 2 h. Centrifuge it at 350–500 g for 5 min, discard the supernatant, and gently tap dry on paper towels. Add 200 µL of 1×Wash Buffer to each well, pipette to resuspend, and incubate for 1 min. After centrifugation and discarding the supernatant, add 25 µL of Detection Antibodies to each well, seal the plate and shake at 800 rpm to incubate at room temperature for 1 h. Add 25 µL of SA‐PE directly to each well, seal the plate and shake at 800 rpm to incubate at room temperature for 30 min. After centrifugation and discarding the supernatant, add 200 µL of 1×Wash Buffer to each well, pipette to resuspend, and incubate for 1 min. After centrifugation and discarding the supernatant, add 200–300 µL of 1×Wash Buffer to each tube for resuspension. Analyze the cell suspension using a Cytek Aurora full spectrum flow cytometer (Cytek Biosciences).

### 4.12 Enzyme Linked Immunosorbent Assay (Elisa)

H23 cell line and LC10 organoids derived from clinical tissue and her pleural fluid were incubated with LM for 24 h, followed by freezing in LN_2_ for 10 s. After adding fresh medium, they were cultured at 37°C for 24 h. Then the supernatants were collected by centrifugation and subjected to HMGB1 quantification using the Human/Mouse HMGB1 ELISA Kit (Proteintech) according to the manufacturer's instructions. Absorbance was measured at 450 nm with a reference wavelength of 630 nm using a multimode microplate reader (EnVision‐II, PerkinElmer). Data were analyzed using ElisaCalc software.

### 4.13 Quantitative PCR (qPCR) Analysis

Total RNA from clinical LC9 and cell lines was extracted using the RNA Easy Fast Tissue/Cell Kit (TIANGEN). Reverse transcription was then performed using the Evo M‐MLV RT Mix Kit (ACCURATE BIOLOGY) under the following conditions: 42°C for 2 min to remove DNA using gDNA Clean Reaction Mix Ver.2, 37°C for 15 min, 85°C for 5 s followed by 4°C incubation to generate cDNA. cDNA was diluted and amplified using the PowerUp SYBR Green Master Mix (ThermoFisher, USA) under the following conditions: 95°C for 2 min followed by 95°C for 3 s and 60°C for 30 s for a total of 40 cycles. Primers used in this study were summarized in Table S5. Target gene expression was normalized with the GAPDH or β‐actin expression level using the 2^−ΔΔCt^ method.

### 4.14 Immunofluorescence of CRT

Cells incubated with LM were snap‐frozen in LN_2_ for 10 s, and cell surface calreticulin (CRT) was detected within 0–4 h. Cells were fixed with 4% paraformaldehyde (PFA) at room temperature for 30 min and washed by phosphate‐buffered saline containing 0.2% Tween (PBST) for 3 times, then blocked with PBST containing 10% FBS at room temperature for 1 – 2 h. PE‐conjugated anti‐CRT antibody (CST) was diluted at 1:300 and incubated overnight at 4°C. After washing to remove residual antibody, cells were stained with DAPI for 5 min and washed three times. and photographed by confocal microscope (Nikon AXR NSPARC).

### 4.15 Flow Cytometry of CRT

Cells were dissociated into single‐cell suspensions using trypsin (Gibco), washed twice with FACS buffer (PBS supplemented with 2% FBS), and resuspended in 100 µL of FACS buffer. Fc receptor was blocked by adding 5 µL of Fc block (eBioscience) and incubating at room temperature for 10 min. Cells were then stained with PE‐conjugated anti‐CRT antibody (1:100 dilution) at 4°C for 1 – 2 h. After washed 2 times, the cell suspension was analyzed using Cytek Aurora full spectrum flow cytometer (Cytek Biosciences).

### 4.16 Culture and Passaging of Cell Line

MC38 cells were cultured in DMEM basic medium (Gibco, United States) and H23 cells were cultured in RPMI 1640 medium (Gibco) with 10% fetal bovine serum (FBS, Gibco, United States) and 1% penicillin‐streptomycin (Gibco, United States) and change the medium every 2 days. Cell line within 10 passages. When the cell density reaches 80–90%, passage the cells. Discard the medium and wash culture dishes with PBS to remove residual medium. Add an appropriate amount of trypsin‐EDTA solution in the dishes, and place it in the incubator for digestion for 2–3 min until cells detach from the dishes. Add pre‐warmed medium and gently pipette to disperse the cells and terminate digestion. Centrifuge to collect the cell pellet, and resuspend 1/3 of the cells to new culture dishes. Place dishes in the incubator after mixing.

### 4.17 Cell Cycle Analysis

To verify the toxicity of LM particles, we examined whether cell proliferation would be affected by unfrozen LM particles. The cell cycle of the MC38 cell line of control, Ga, and Bi‐Ga groups were detected using the BeyoClick EdU Cell Proliferation Kit (Beyotime Biotechnology). After the cell line adhered to the well, LM particles were added at a volume of 1/100 and incubated for two days to allow the cells to reach a suitable density. Then, the operation was performed according to the instructions. The EdU working solution was diluted to 10 mM with culture medium and preheated. Add 2 mL per well EdU working solution to the six‐well plate of the MC38, and incubate at 37°C for 2 h. After digestion of cells by Trypsin, 1 mL of 4% PFA was added to the collected cells for fixation at room temperature for 15 min. Then the cells were washed 3 times with washing solution (PBS containing 3% BSA), each time for 3–5 min. Add 1 mL of permeabilization buffer (PBS containing 0.3% Triton X‐100) and incubate at room temperature for 10–15 min. Wash with washing buffer 1–2 times. Strictly follow the order and volume to prepare the total Click reaction solution required for all groups in the experiment, in which the cells in one 6‐well plate require 430 µL Click Reaction Buffer, 20 µL CuSO4, 1 µL Azide 488, and 50 µL Click Additive Solution, for a total of 500 µL. Add the Click reaction solution to the permeabilized cells and incubate at room temperature in the dark for 30 min. After washing 3 times, dilute Hoechst 33342 at 1:1000, 1 mL/well, and incubate at room temperature for 10 min. After washing three times, analyze the cell suspension using a Cytek Aurora full spectrum flow cytometer (Cytek Biosciences), and establish corresponding single‐stained and negative controls. The collected data are then analyzed using FlowJo v.10.10.0 software.

### 4.18 Bulk RNA Sequencing

Sequencing was performed using previously published methods 26. Organoids on the InSMAR‐chip were digested in situ using TripLE (Gibco). Two oligo P1 and P2 were ligated into complete barcode using T4 DNA Ligase (NEB) and a linker. The primers and P2 sequences are in Table S5. There are 96 barcodes, because there are 96 P2, so different types and different treatment conditions of organoids can be distinguished in a library generated by a single InSMAR‐chip, greatly reducing sequencing costs. Barcodes, Dynabeads M‐270 (Invitrogen), and lysis buffer were respectively pipetted into the chip using an Echo 650 acoustic liquid handling system (BECKMAN COULTER), and the beads and barcodes were ligated to capture mRNA. The lysis buffer was made by dissolving 0.075 g Triton X‐100 (YEASEN) into 50 mL 1× RT buffer (pH 8.0, Thermo Scientific). After the mRNA released by cell lysis was captured by magnetic beads, it was recovered and washed with 6X SSC solution. Maxima H Minus Reverse Transcriptase reagent (Thermo Scientific), template‐switching TSO block, dNTP, RNase inhibitor, etc. were added and incubated at room temperature for 30 min, and then incubated at 42°C for 90 min to obtain cDNA. Then, 2×HotStart Readymix (KAPA Biosystems), P7 primer (10 µM) and TSO primer (10 µM) were added for PCR. DNA was fragmented to 300 bp by Covaris M220 (Covaris). Subsequently, end repair, 5' phosphorylation, DA‐Tailing, adaptor ligation, size selection, and PCR enrichment were performed sequentially using NEBNext Ultra II DNA Library Prep Kit for Illumina (NEB). The product was purified using AMPure XP beads (BECKMAN), and the quality of the library was controlled using Agilent Bioanalyzer 4200. mRNA libraries and paired‐end 150 bp (PE150) sequencing were done by Novogene, China. The sequencing data were processed via the Galaxy online platform. Specifically, after trimming adaptor sequences from the paired‐end reads, alignment was performed against the human reference genome GRCh38 (hg38), and feature counts were subsequently generated. Differential gene expression analysis was carried out through the DESeq2 tool, with differentially expressed genes (DEGs) being defined as those meeting the criteria of False discovery rate (FDR) < 0.05 and absolute log2 fold change > 1. Gene enrichment analysis was then conducted using these DEGs through the g: Profiler online tool, and the results were visualized with Cytoscape software [54], based on the gene ontology (GO) and Kyoto Encyclopedia of Genes and Genomes (KEGG) databases.

### 4.19 Statistical Analysis

Microsoft Excel and GraphPad Prism 10 were used for data analysis and plotting. The differences between groups were analyzed by one way analysis of variance (ANOVA) for all P values. Two—way ANOVA was used to assess the individual and interactive effects of Matrigel and LM on organoids viability. (*p < 0.05, **p < 0.01, ***p < 0.001, ****p < 0.0001, and *p* value lower than 0.05 is considered significantly different; ns, not significant). Values in column bar plots were illustrated as mean ± Standard Error of the Mean (SEM). Flow cytometry data were analyzed with FlowJo v.10.10.0 software. Imaris 9.9 and ImageJ were used for image signal processing and quantitative statistics. ElisaCalc was used for Elisa data processing with four‐parameter logistic (4‐PL) curve fitting.

## Funding

Beijing Natural Science Foundation (No. L245017); National Natural Science Foundation of China (No. 82502578, No. 82472173); the Capital Health Research and Development of Special (No. 12024B02002); and Beijing Tsinghua Changgung Hospital Fund (No. 12023C01009).

## Conflicts of Interest

The authors declare no conflicts of interest.

## Supporting information




**Supporting File 1**: advs75172‐sup‐0001‐FigureS1–S28.docx.

**Supporting File 2**: advs75172‐sup‐0002‐TableS1–S10.xlsx.

**Supporting File 3**: advs75172‐sup‐0003‐VideoS1.mp4.

**Supporting File 4**: advs75172‐sup‐0004‐VideoS2.mp4.

**Supporting File 5**: advs75172‐sup‐0005‐VideoS3.mp4.

**Supporting File 6**: advs75172‐sup‐0006‐VideoS4.mp4.

## Data Availability

The data that support the findings of this study are available from the corresponding author upon reasonable request.
