# Supplementary material for: Liquid Metal Nanotransformers for Drug‐Resistant Pan‐Cancer Therapy in Patient‐Derived Organoids
Source: Adv Sci (Weinh). 2026 Apr 13;13(36):e21041. doi: 10.1002/advs.202521041 (PMC13317767; doi:10.1002/advs.202521041)
Supplement: Supplementary file 1 — Supporting File 1: advs75172‐sup‐0001‐FigureS1–S28.docx. [file ADVS-13-e21041-s005.docx]

Supporting Information

Liquid Metal Nanotransformers for Drug-Resistant Pan-Cancer Therapy in Patient-Derived Organoids

Xiaojie Yuan, Xuelin Wang^*^, Zhongyao Chen, Shuo Wang, Weining Gao, Ziyi Chen, Runyang Li, Cheng Hao, Lei Zhang, Qiongqiong Zhang, Ke Zhang, Mei Yu, Qian Liu, Jingxuan Wang, Huiping Li, Simpkins Fiona, Jing Liu, Qiang Liu^*^, Peng Liu^*^, and Yawei Hu^*^


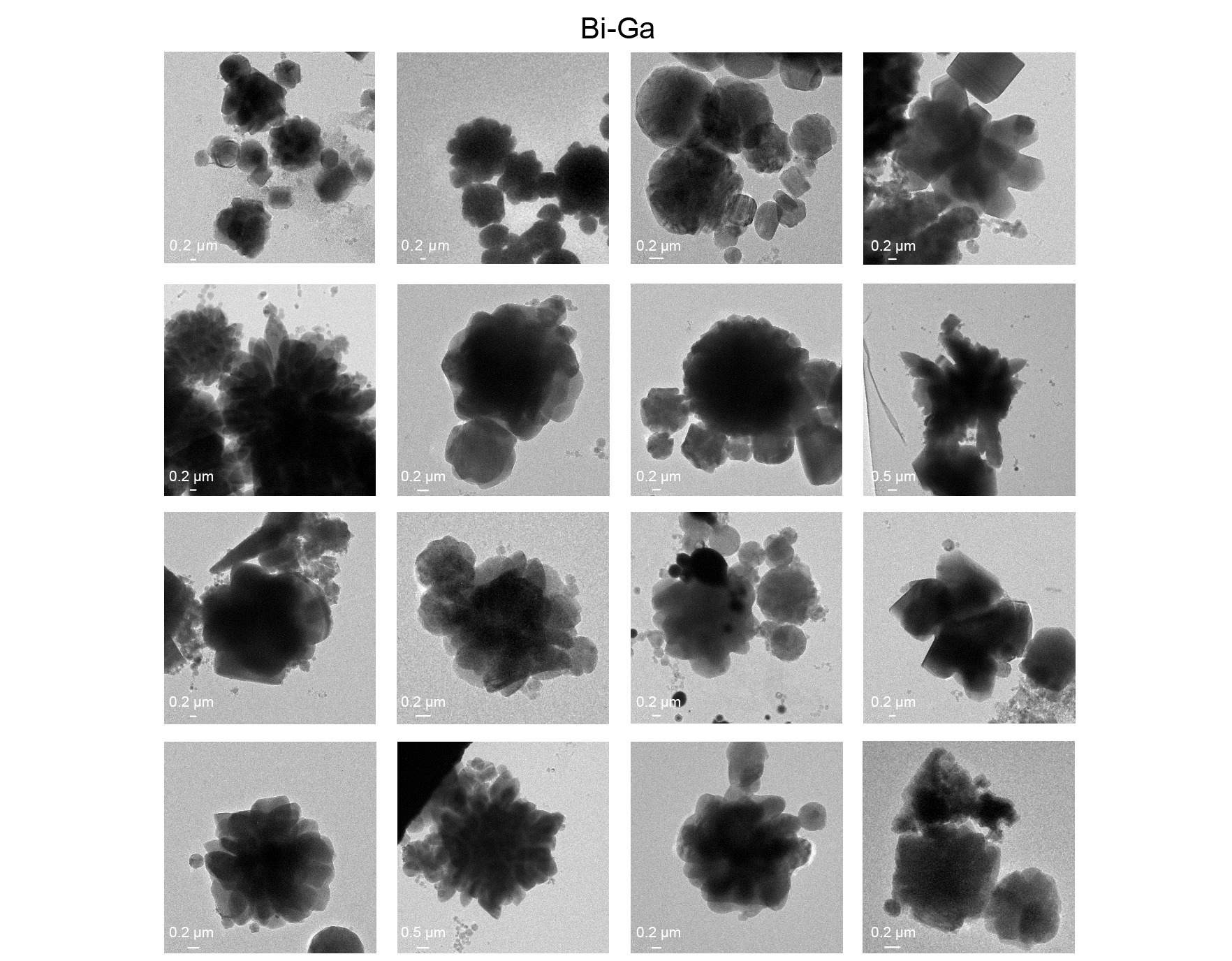


**Figure S1.** Cryo-TEM images of Bi-Ga particles under freezing treatment.


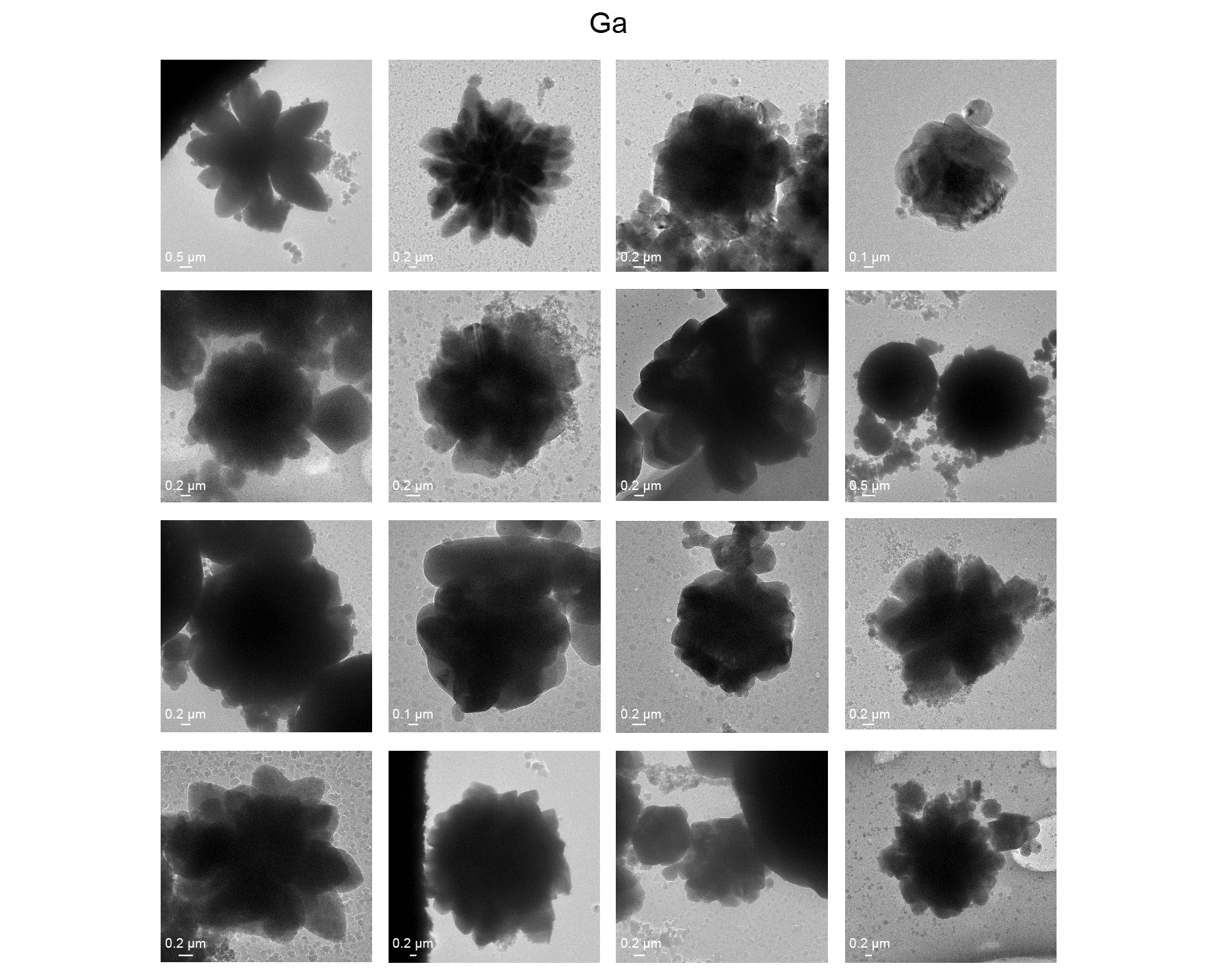


**Figure S2.** Cryo-TEM images of Ga particles under freezing treatment.


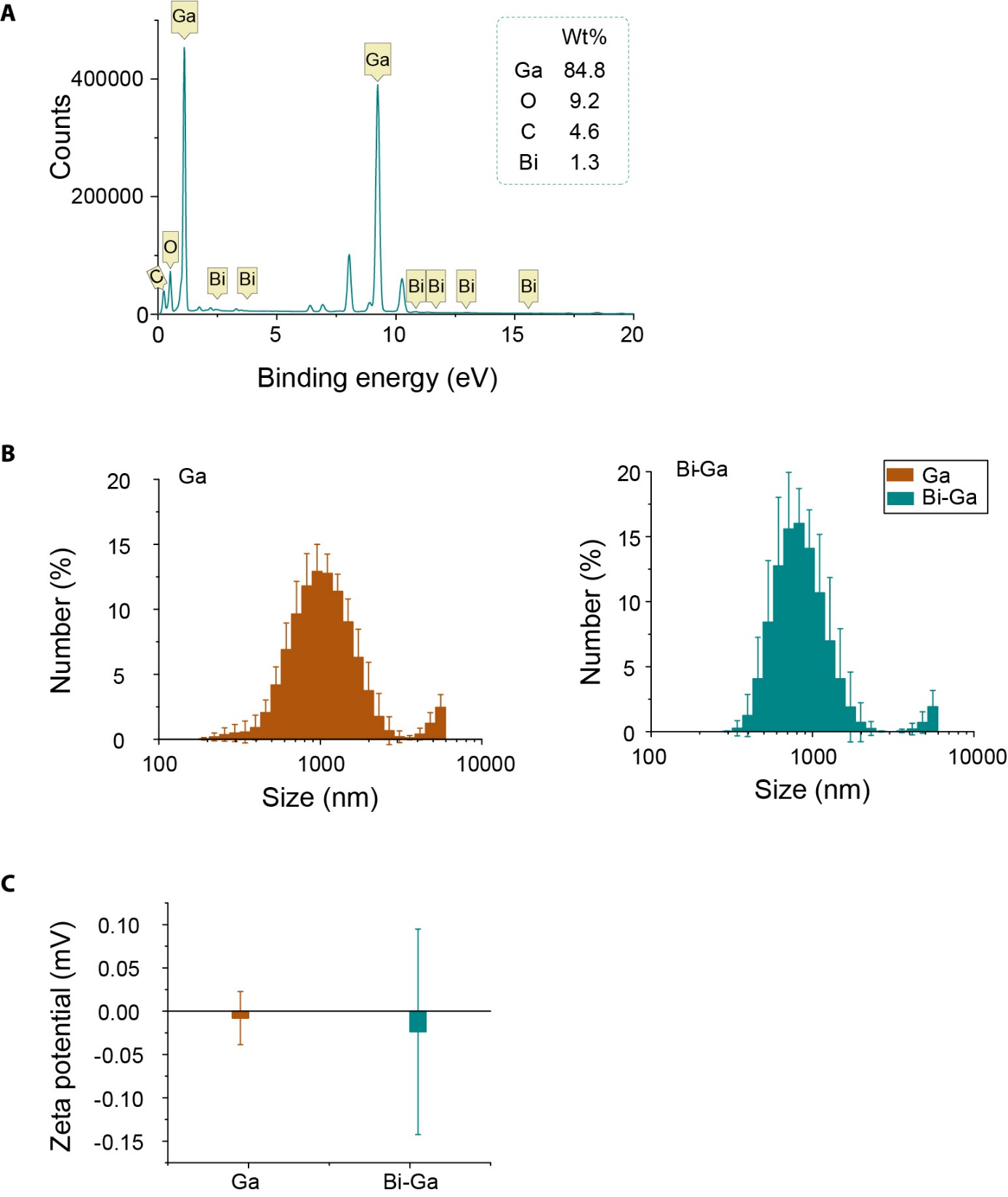


**Figure S3.** Characterization of LM properties. (A) EDS analysis of transformed Bi-Ga particle. (B) The hydrodynamic size of Ga and Bi-Ga particles. (C) Zeta potential of Ga and Bi-Ga particles.


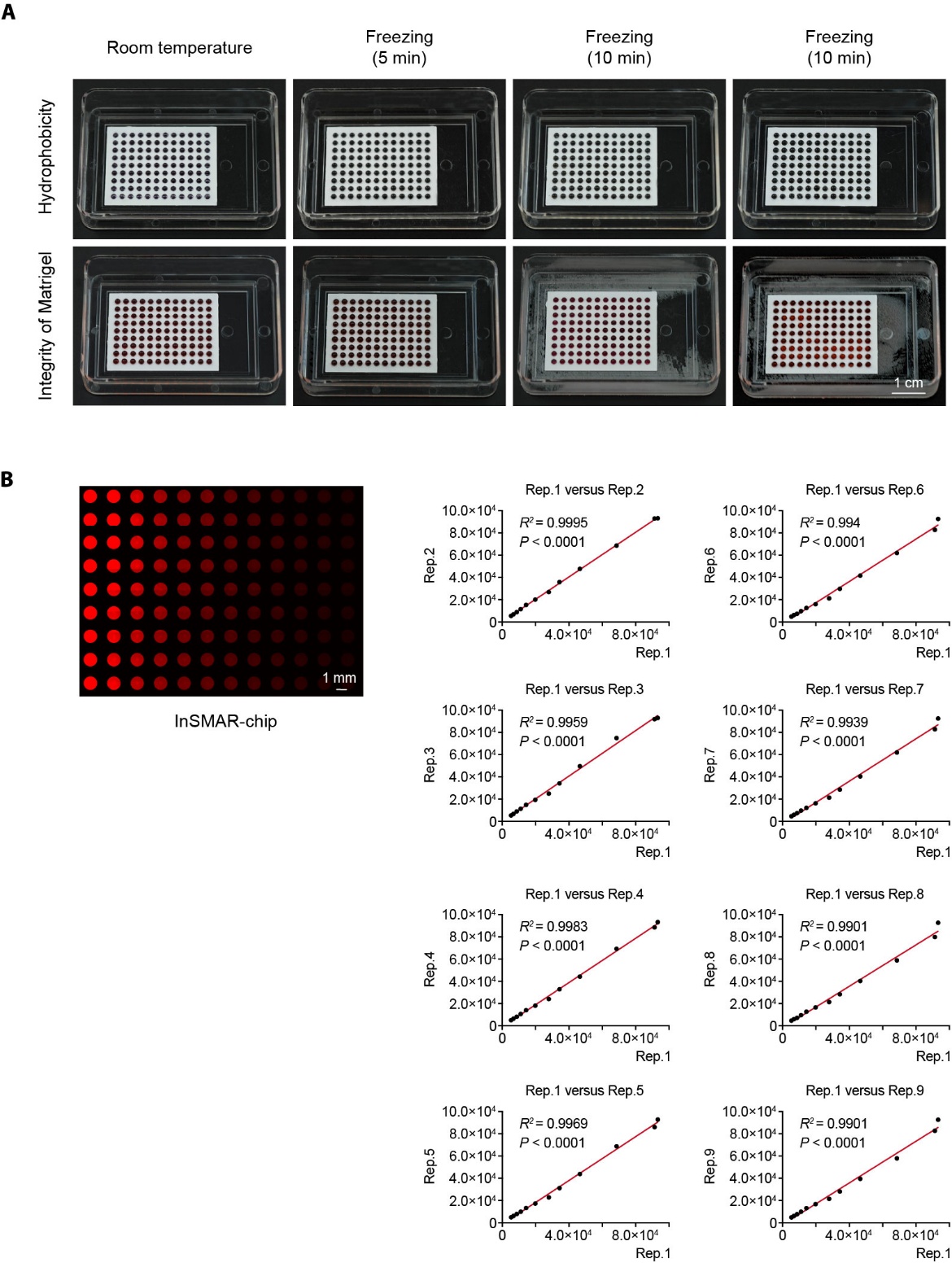


**Figure S4.** Performance of InSMAR-chip after freezing for 5, 10, and 20 min. (**A**) Evaluation of InSMAR-chip surface hydrophobicity and the integrity of Matrigel after freezing on liquid nitrogen for 5-, 10-, and 20-min. Scale bar: 1 cm. (**B**) After freezing fluorescence scanning showed intensity patterns consistent with the gradient dye solution added in microwells on the chip. The fluorescence intensity across different rows exhibited a correlation coefficient > 0.99. Scale bar: 1 mm.


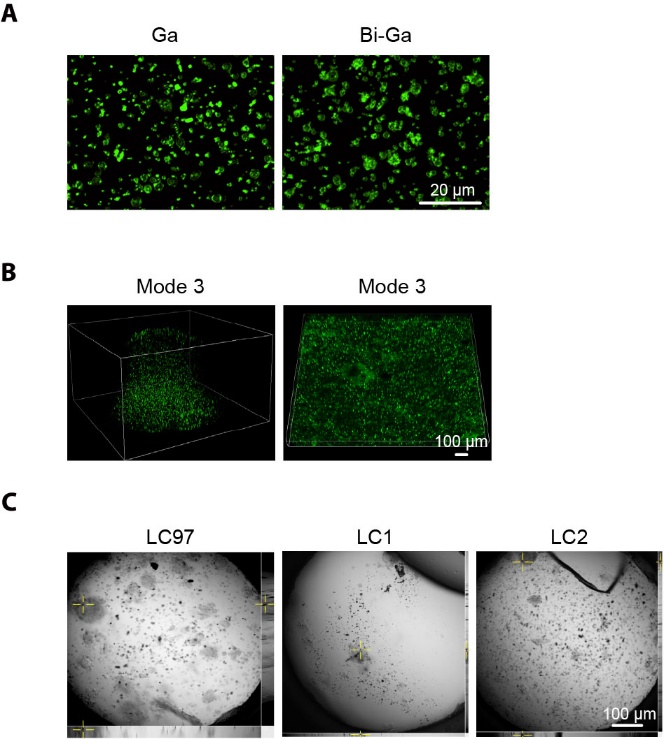


**Figure S5**. LM particles enter organoids after co-incubation. (**A**) Confocal fluorescence images of Ga and Bi-Ga particles. Scale bar: 20 μm. (**B**) Three-dimensional confocal reconstruction images of Bi-Ga with fluorescence falling with gravity in Matrigel of Mode 3. Bi-Ga surrounded and entered the organoids 3 days after inoculation. Scale bar: 100 μm. (**C**) The projection positions of LM particles in LC97, LC1, LC2 organoids after 24 h of incubation in Mode 3. LM particles were observed both within and surrounding the organoids.


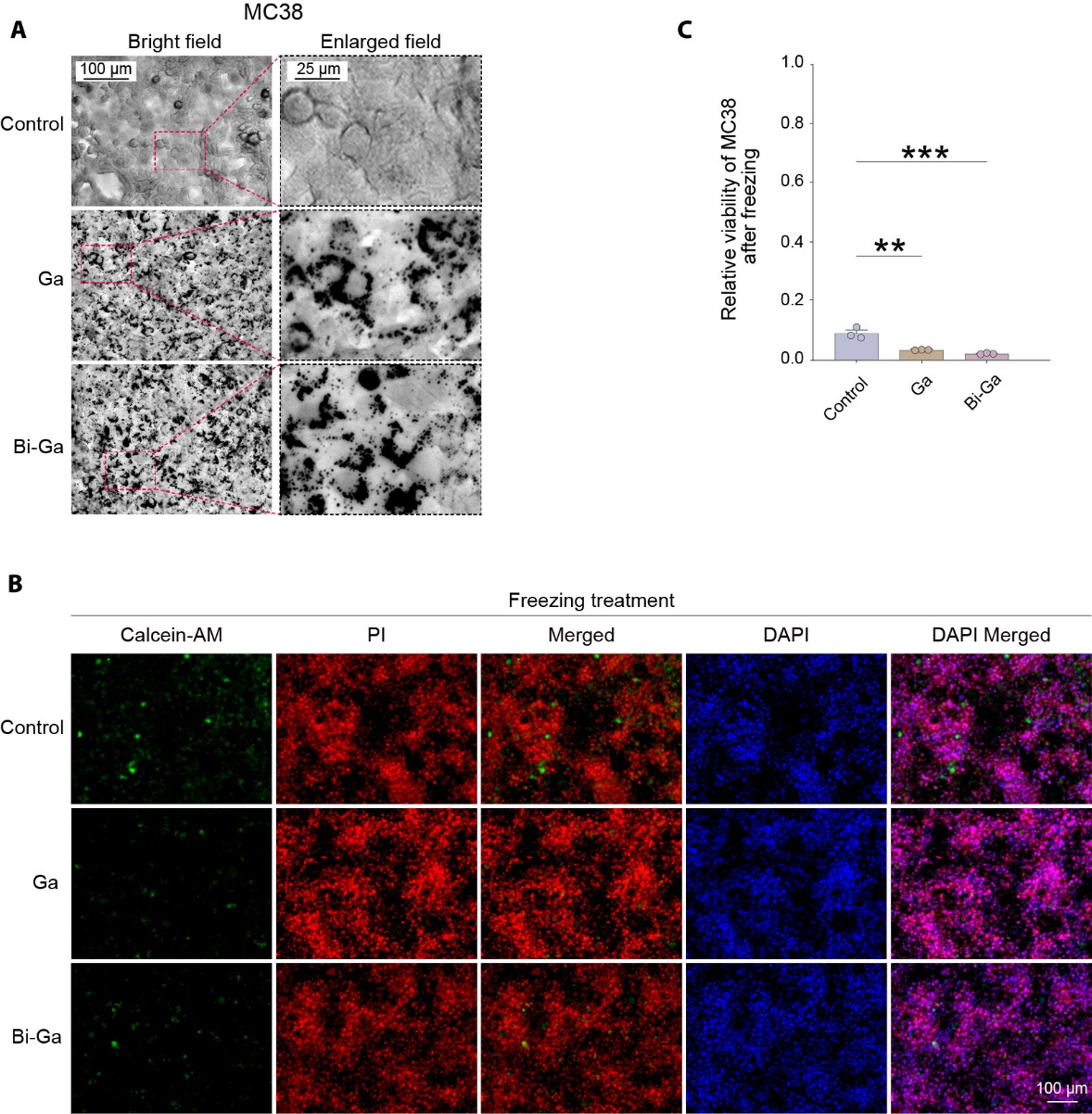


**Figure S6.** Endocytosis and killing effect after freezing of LM particles in MC38 cells. (**A**) Endocytosis of Ga, Bi-Ga by MC38 cells after 4 h of incubation compared with the control group. (**B**) Fluorescence images of MC38 cells following cryo-treatment under three conditions: Control, Ga, Bi-Ga. Dead cells are stained with propidium iodide (PI, red), live cells with Calcein-AM (green), nuclei with Hoechst (blue). Scale bar: 100 μm. (**C**) Relative viability of MC38 cells in the Control, Ga, Bi-Ga groups after freezing.


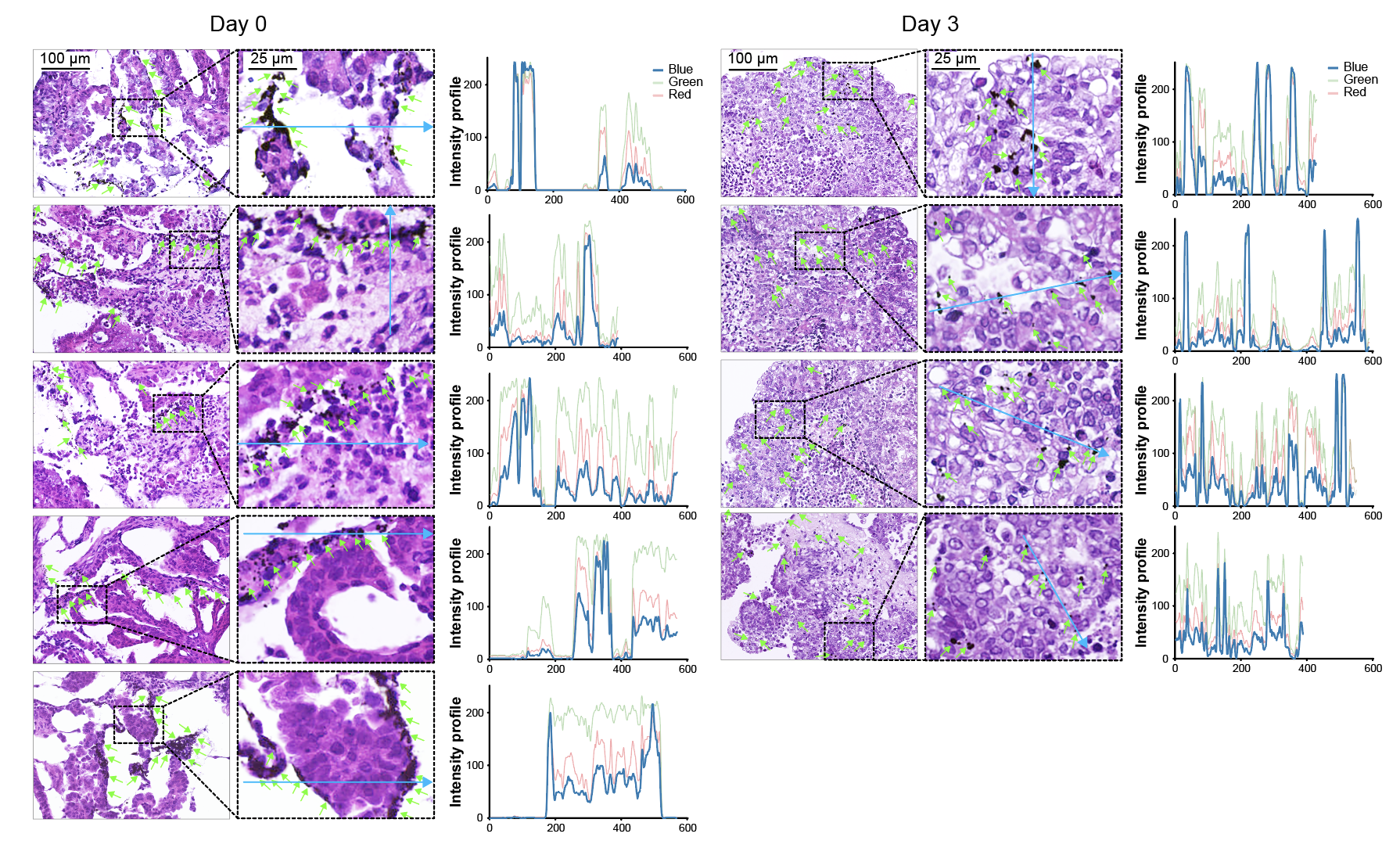


**Figure S7.** Endocytosis of injected LM particles in ovarian cancer tissues. H&E staining sections showing the distribution of LM particles in ovarian cancer tissue immediately after injection and after 3 days of in vitro culture. Green arrows indicate the location of LM particles. The intensity profile shows the pixel intensity distribution of the red, green, and blue channels in the H&E section area through which the blue line passes. Peaks of the blue curve best represents the location of LM particles, while the red and green curves correspond to areas such as the cytoplasm.


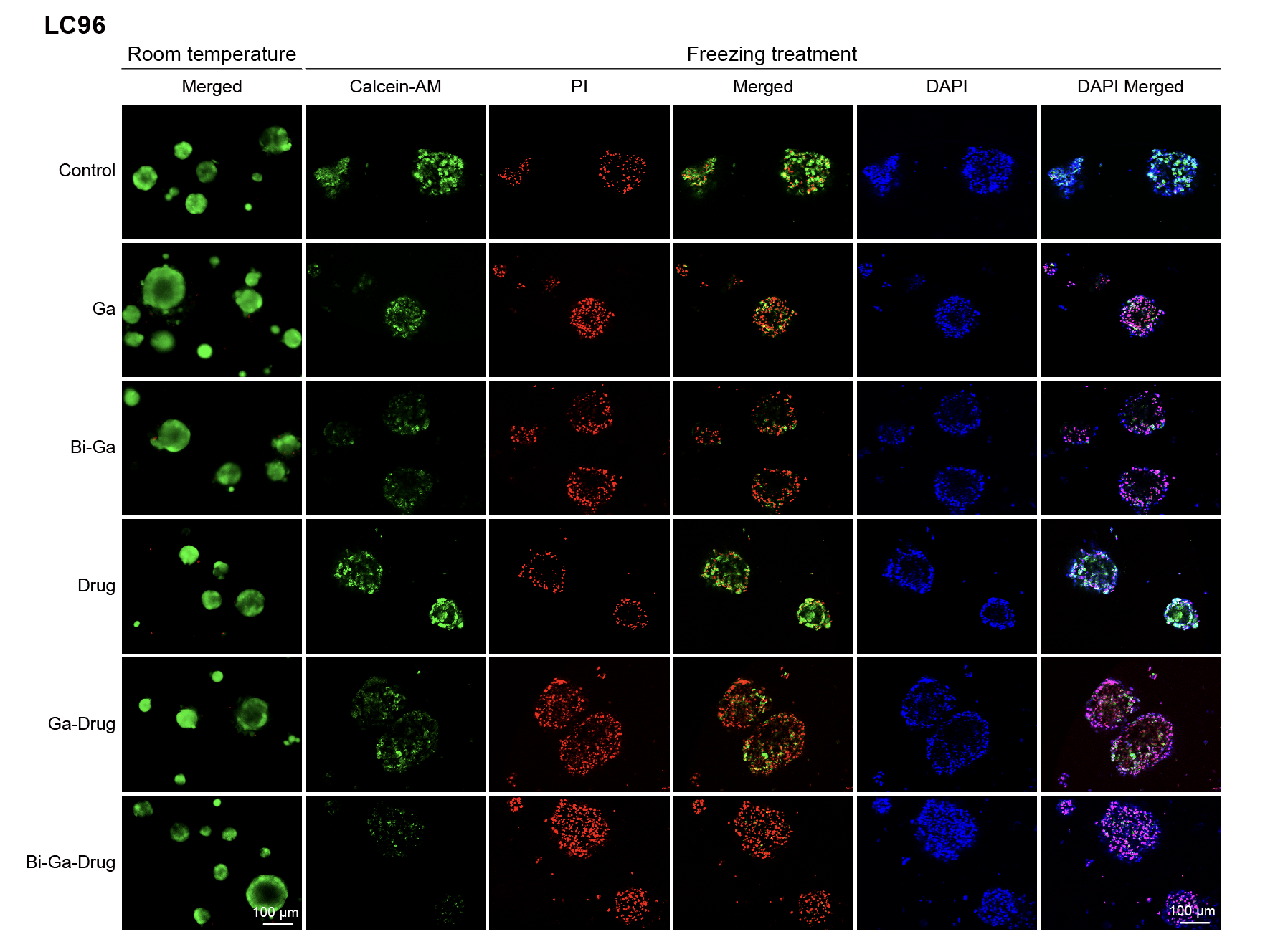


**Figure S8.** LM particles exert killing effect in LC96 organoid line after freezing. Fluorescence images of LC96 organoids, with or without cryo-treatment, under 6 groups of conditions after 3 days (Control, Ga, Bi-Ga, chemotherapeutic drug, Ga combined with chemotherapeutic drug, Bi-Ga combined with chemotherapeutic drug). Dead cells are labeled with PI (red), live cells with Calcein-AM (green), and all cell nuclei with Hoechst (blue). Scale bar: 100 μm.


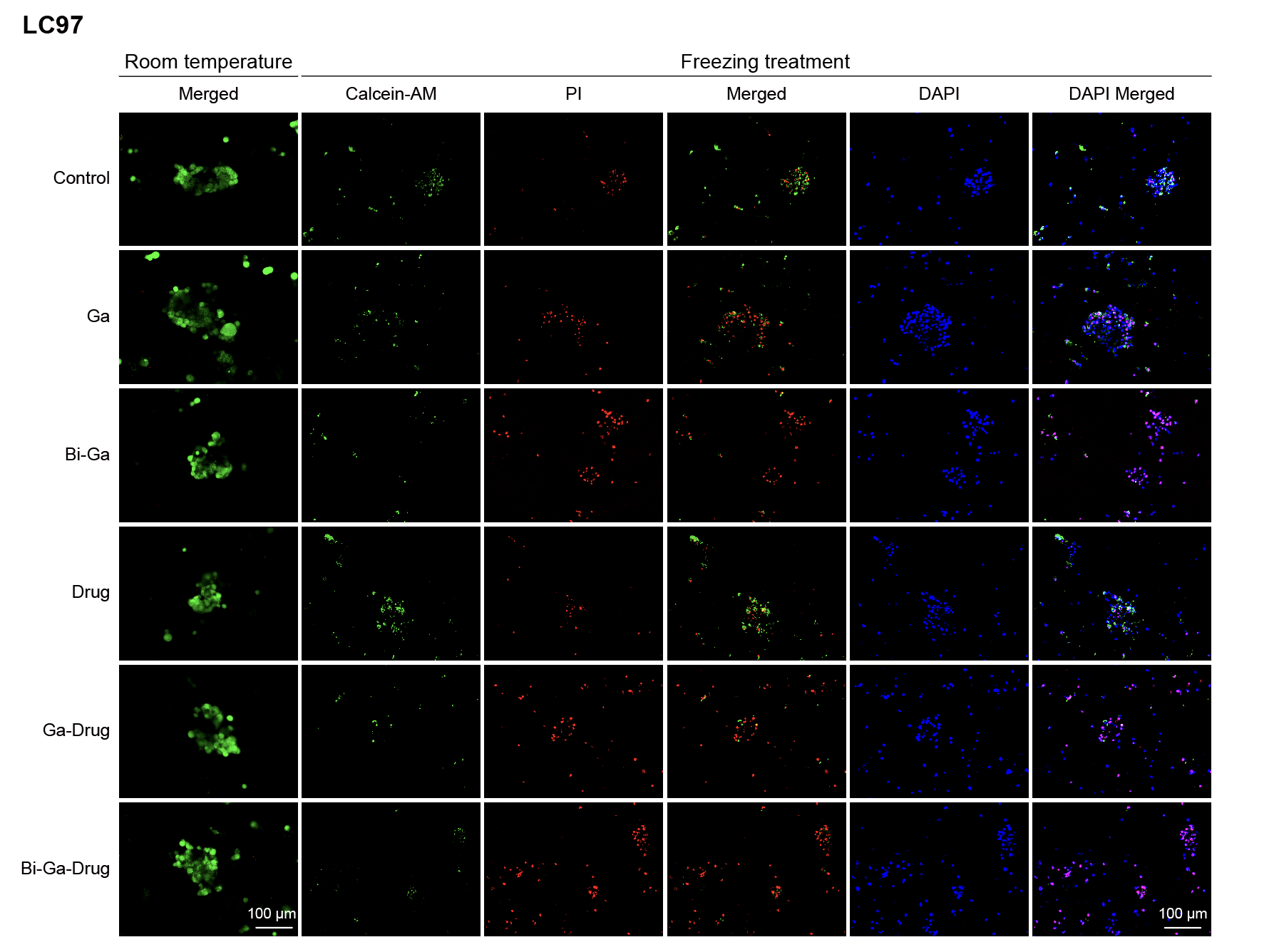


**Figure S9.** LM particles exert killing effect in LC97 organoid line after freezing. Fluorescence images of LC97 organoids, with or without cryo-treatment, under 6 groups of conditions after 3 days (Control, Ga, Bi-Ga, chemotherapeutic drug, Ga combined with chemotherapeutic drug, Bi-Ga combined with chemotherapeutic drug). Dead cells are labeled with PI (red), live cells with Calcein-AM (green), and all cell nuclei with Hoechst (blue). Scale bar: 100 μm.


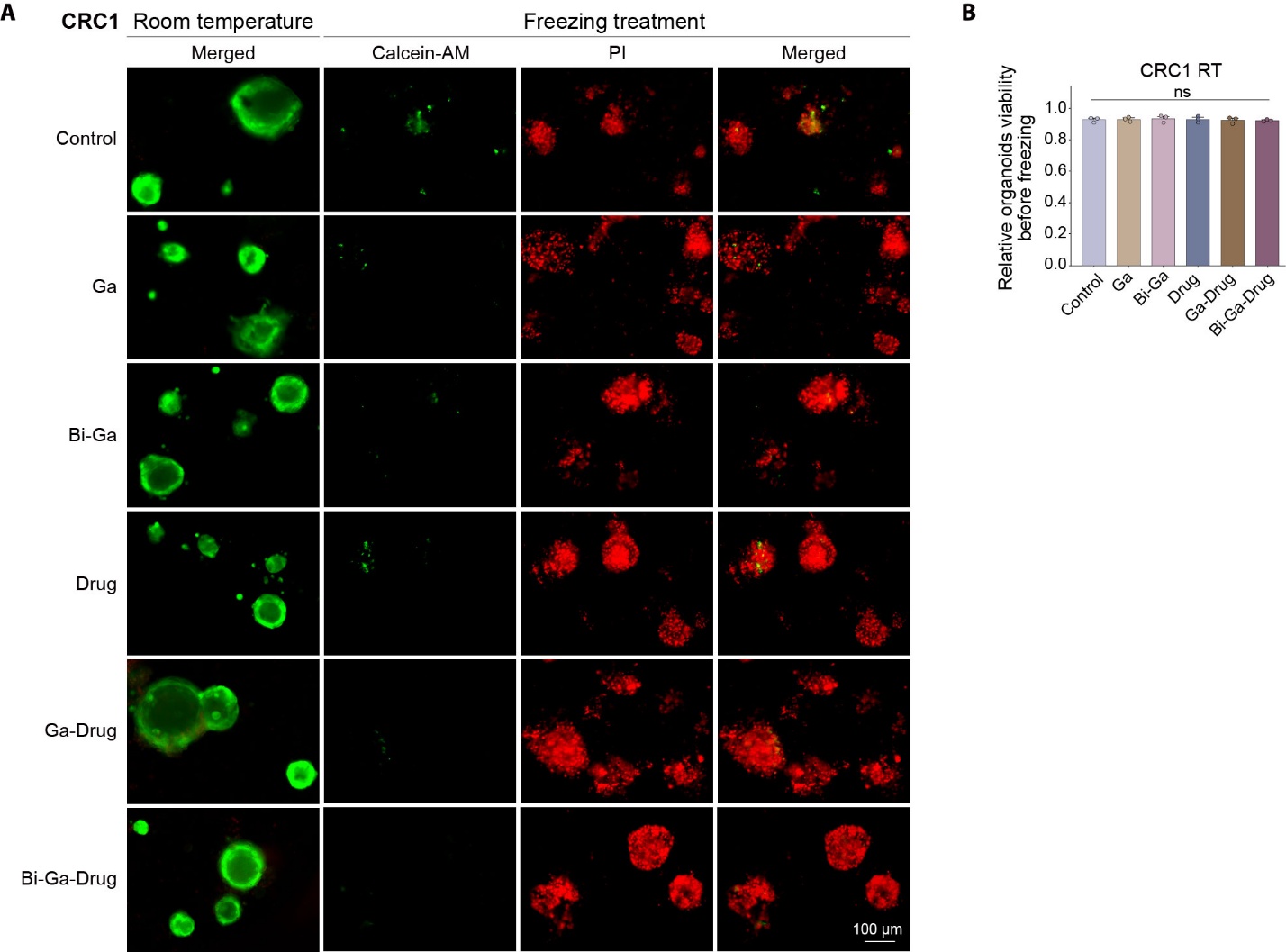


**Figure S10.** LM particles exert killing effect in CRC1 organoid line after freezing. (**A**) Fluorescence images of CRC1 organoids, with or without cryo-treatment, under 6 groups of conditions after 3 days (Control, Ga, Bi-Ga, chemotherapeutic drug, Ga combined with chemotherapeutic drug, Bi-Ga combined with chemotherapeutic drug). Dead cells are labeled with PI (red), live cells with Calcein-AM (green). Scale bar: 100 μm. (**B**) Relative viability of CRC1 at room temperature under the same 6 treatment conditions after 3 days. Experiment was repeated three times. Data are shown as mean ± SEM. Statistical significance was determined by one-way ANOVA (ns, not significant). RT stands for Room Temperature.


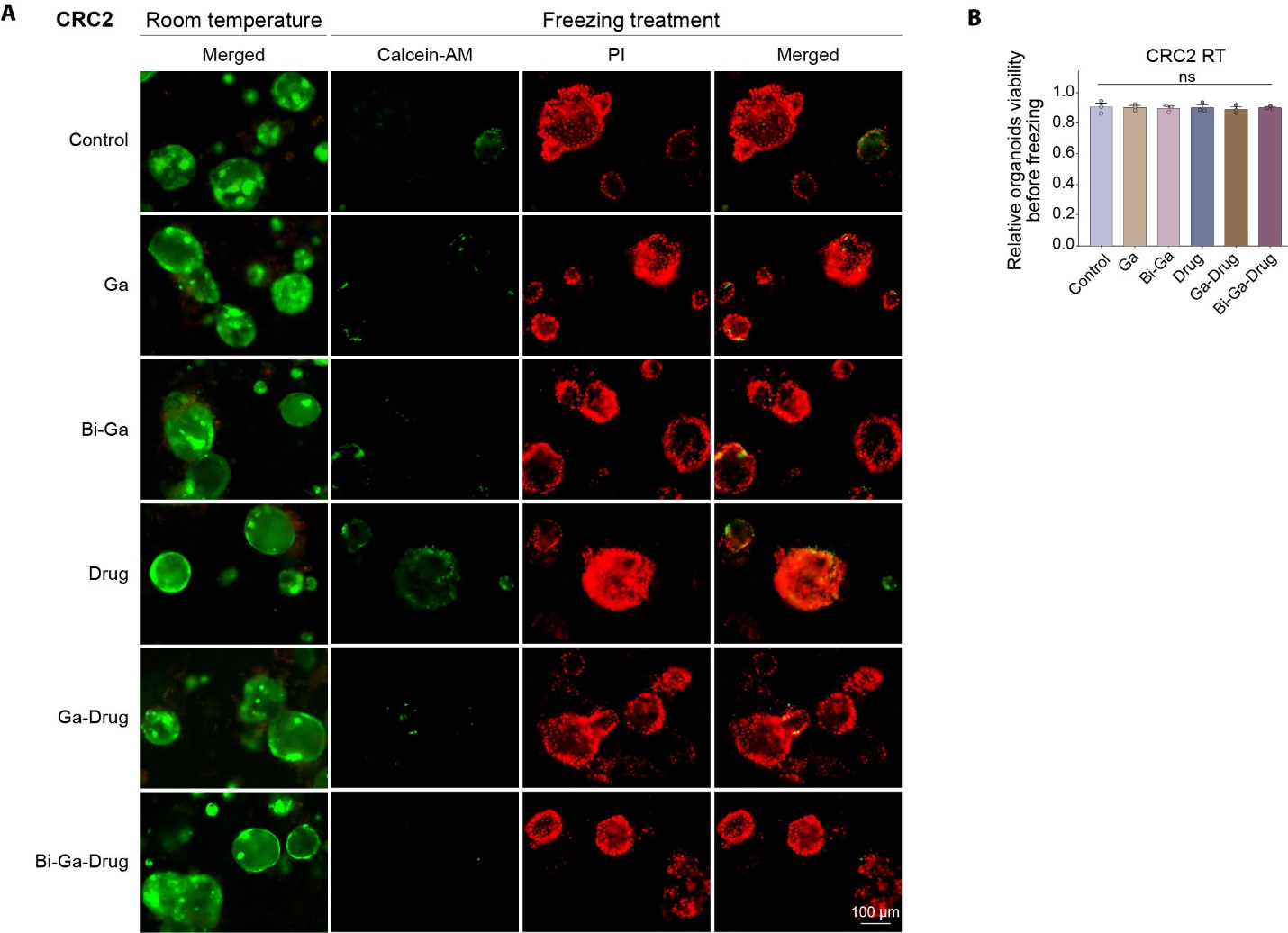


**Figure S11.** LM particles exert killing effect in CRC2 organoid line after freezing. (**A**) Fluorescence images of CRC2 organoids, with or without cryo-treatment, under 6 groups of conditions after 3 days (Control, Ga, Bi-Ga, chemotherapeutic drug, Ga combined with chemotherapeutic drug, Bi-Ga combined with chemotherapeutic drug). Dead cells are labeled with PI (red), live cells with Calcein-AM (green). Scale bar: 100 μm. (**B**) Relative viability of CRC2 at room temperature under the same 6 treatment conditions after 3 days. Experiment was repeated three times. Data are shown as mean ± SEM. Statistical significance was determined by one-way ANOVA (ns, not significant). RT stands for Room Temperature.


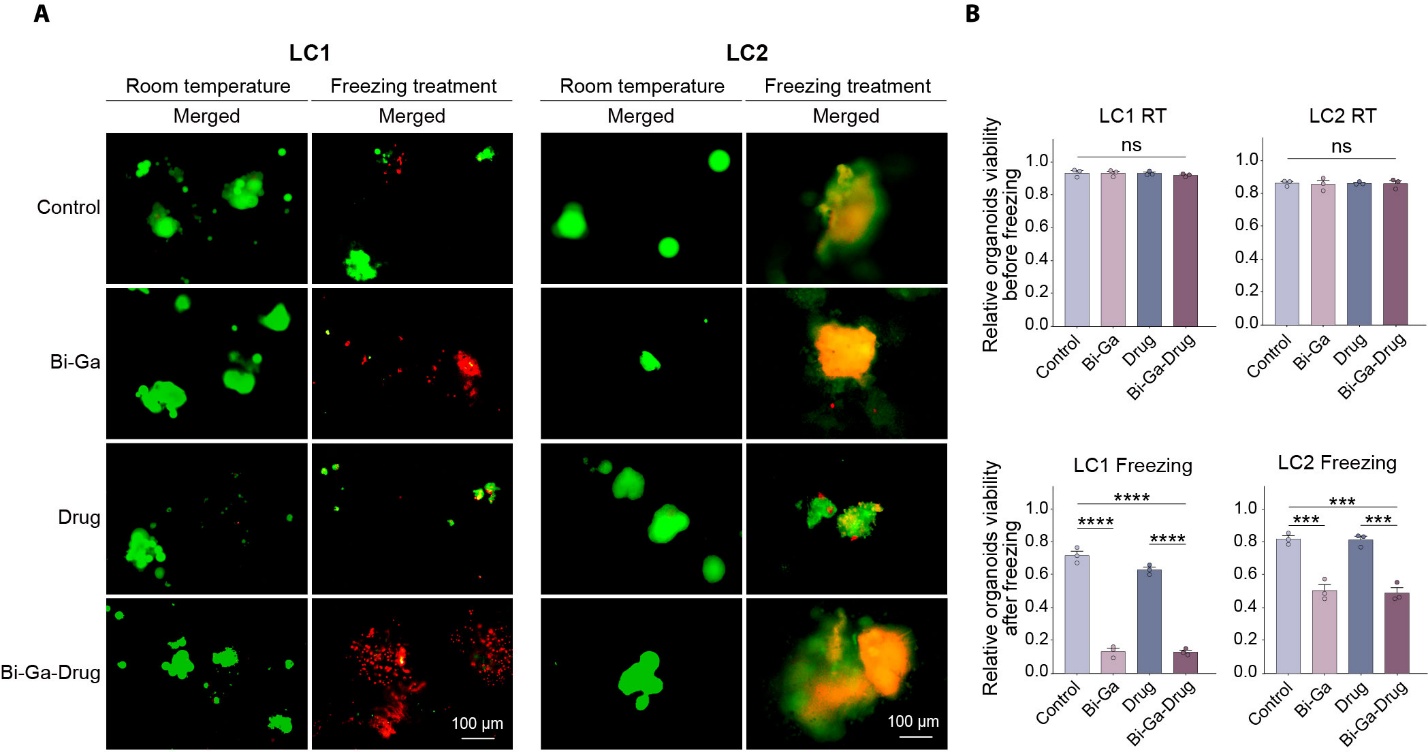


**Figure S12.** LM particles exert killing effect in LC1 and LC2 PDOs after freezing. (**A**) Fluorescence images of LC1 and LC2 organoids, with or without cryo-treatment, under 4 groups of conditions after 3 days (Control, Bi-Ga, chemotherapeutic drug, Bi-Ga combined with chemotherapeutic drug). Dead cells are labeled with PI (red), live cells with Calcein-AM (green). Scale bar: 100 μm. (**B**) Relative viability of LC1 and LC2 organoids under the same 4 treatment conditions in frozen and non-frozen groups. Experiment was repeated three times. Data are shown as mean ± SEM. Statistical significance was determined by one-way ANOVA (ns, not significant). RT stands for Room Temperature.


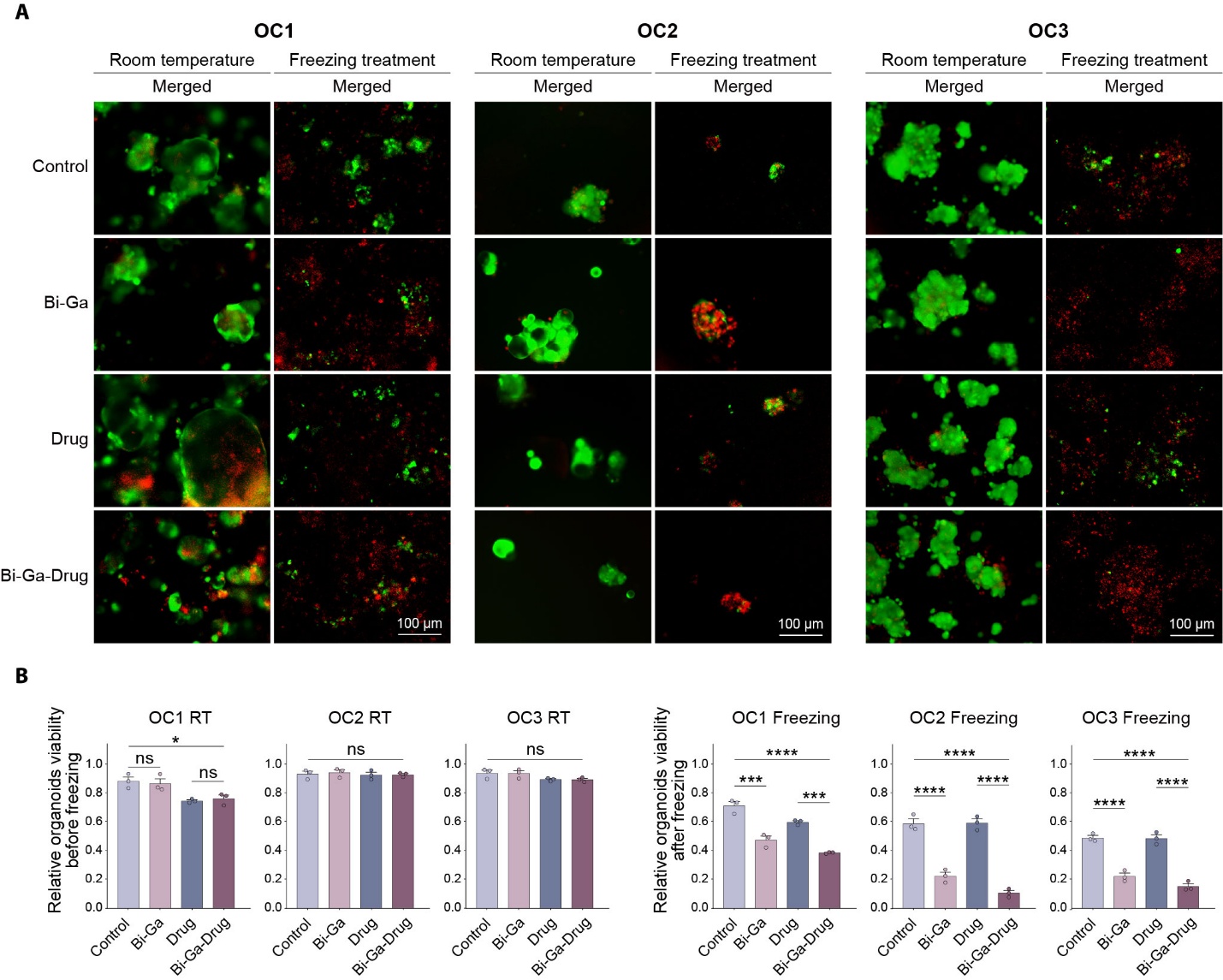


**Figure S13.** LM particles exert killing effect in OC1, OC2 and OC3 PDOs after freezing. (**A**) Fluorescence images of OC1, OC2 and OC3 organoids, with or without cryo-treatment, under 4 groups of conditions after 3 days (Control, Bi-Ga, chemotherapeutic drug, Bi-Ga combined with chemotherapeutic drug). Dead cells are labeled with PI (red), live cells with Calcein-AM (green). Scale bar: 100 μm. (**B**) Relative viability of OC1, OC2, and OC3 organoids under the same 4 treatment conditions in frozen and non-frozen groups. Experiment was repeated three times. Data are shown as mean ± SEM. Statistical significance was determined by one-way ANOVA (ns, not significant). RT stands for Room Temperature.


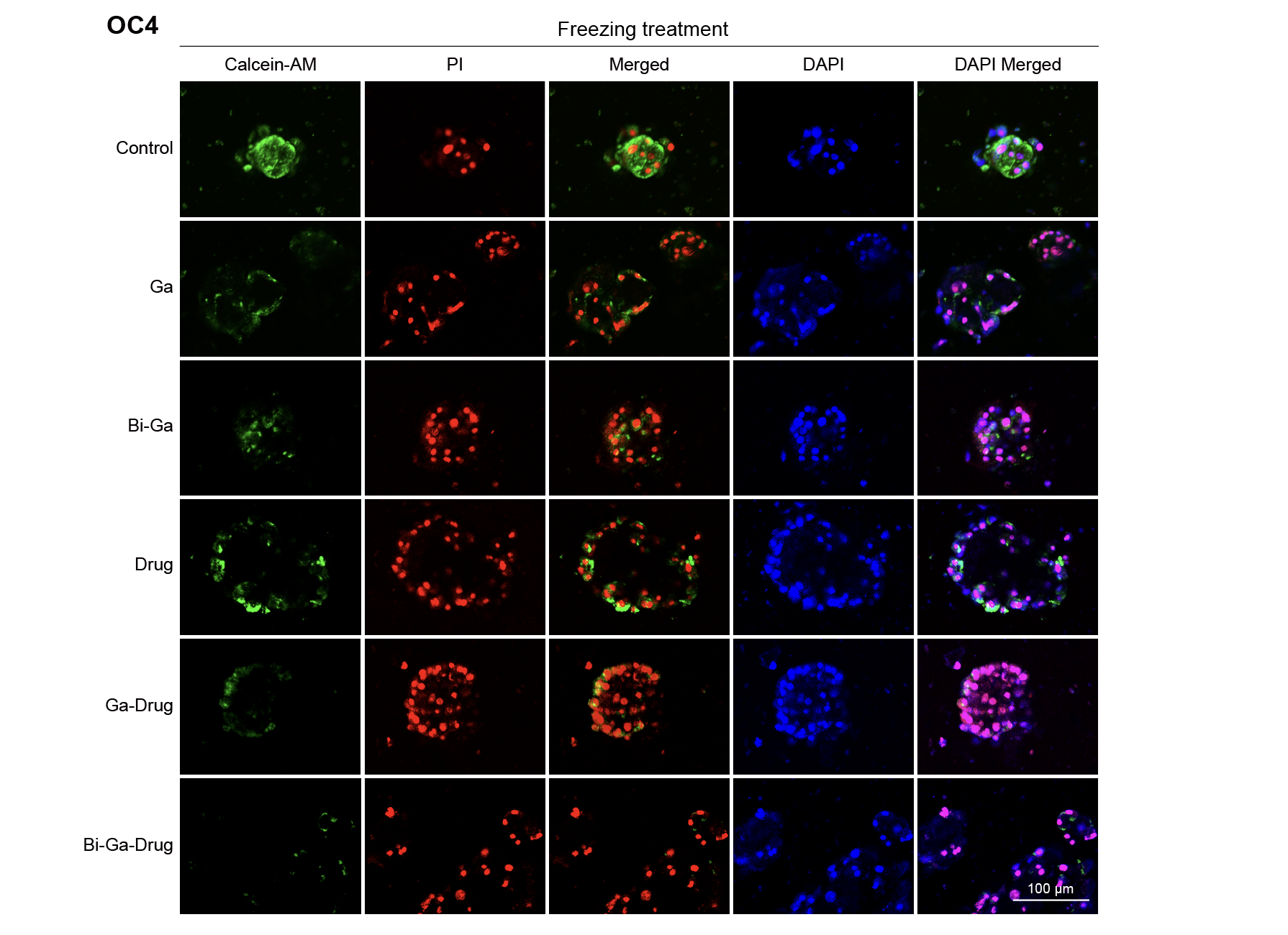


**Figure S14.** LM particles exert killing effect in OC4 PDOs after freezing. Fluorescence images of OC4 organoid after freezing treatment under 6 groups of conditions after 3 days: Control, Ga, Bi-Ga, chemotherapeutic drug, Ga combined with chemotherapeutic drug, Bi-Ga combined with chemotherapeutic drug. Dead cells are labeled with PI (red), live cells with Calcein-AM (green), and all cell nuclei with Hoechst (blue). Scale bar: 100 μm.


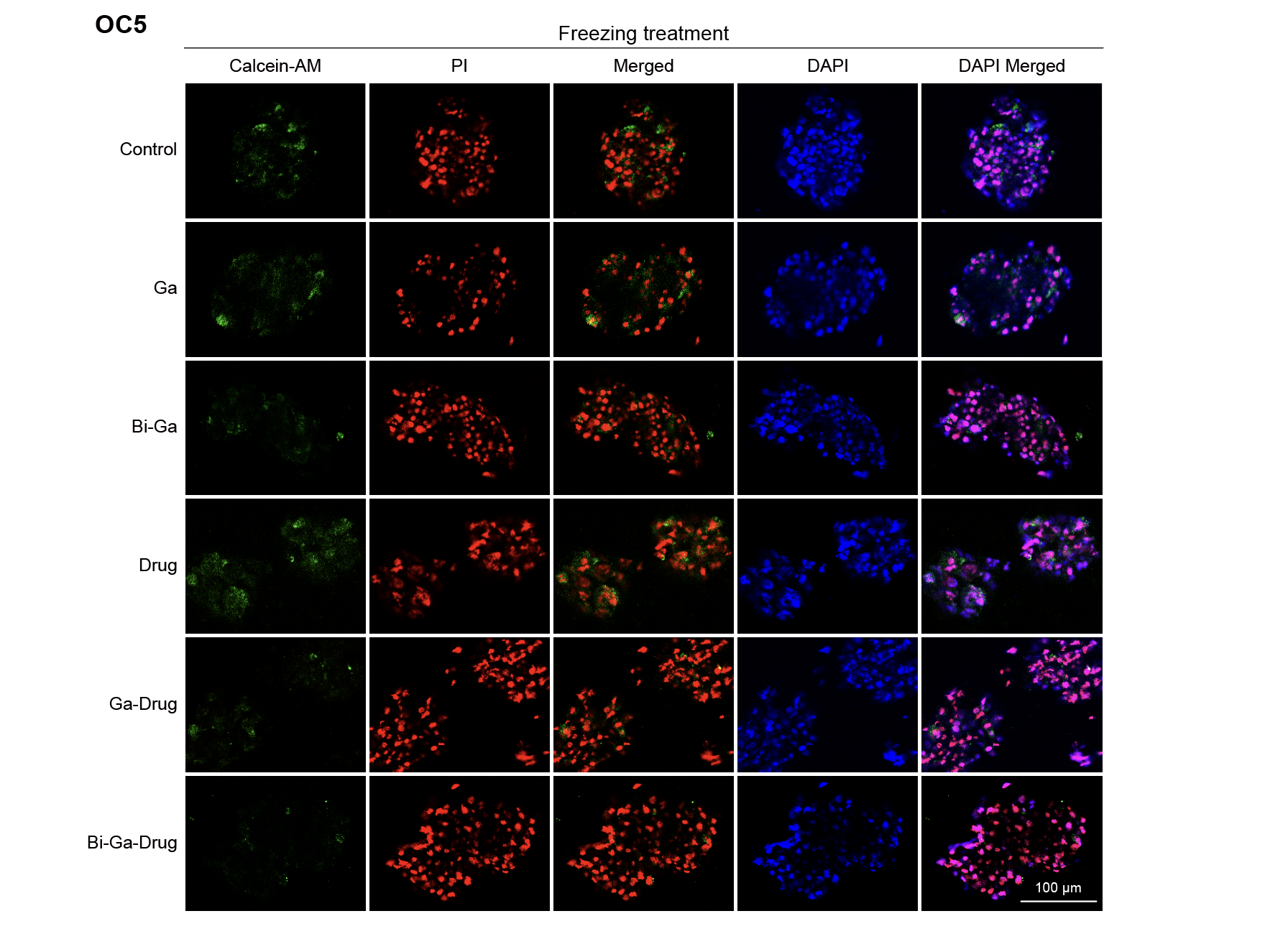


**Figure S15.** LM particles exert killing effect in OC5 PDOs after freezing. Fluorescence images of OC5 organoid after freezing treatment under 6 groups of conditions after 3 days: Control, Ga, Bi-Ga, chemotherapeutic drug, Ga combined with chemotherapeutic drug, Bi-Ga combined with chemotherapeutic drug. Dead cells are labeled with PI (red), live cells with Calcein-AM (green), and all cell nuclei with Hoechst (blue). Scale bar: 100 μm.


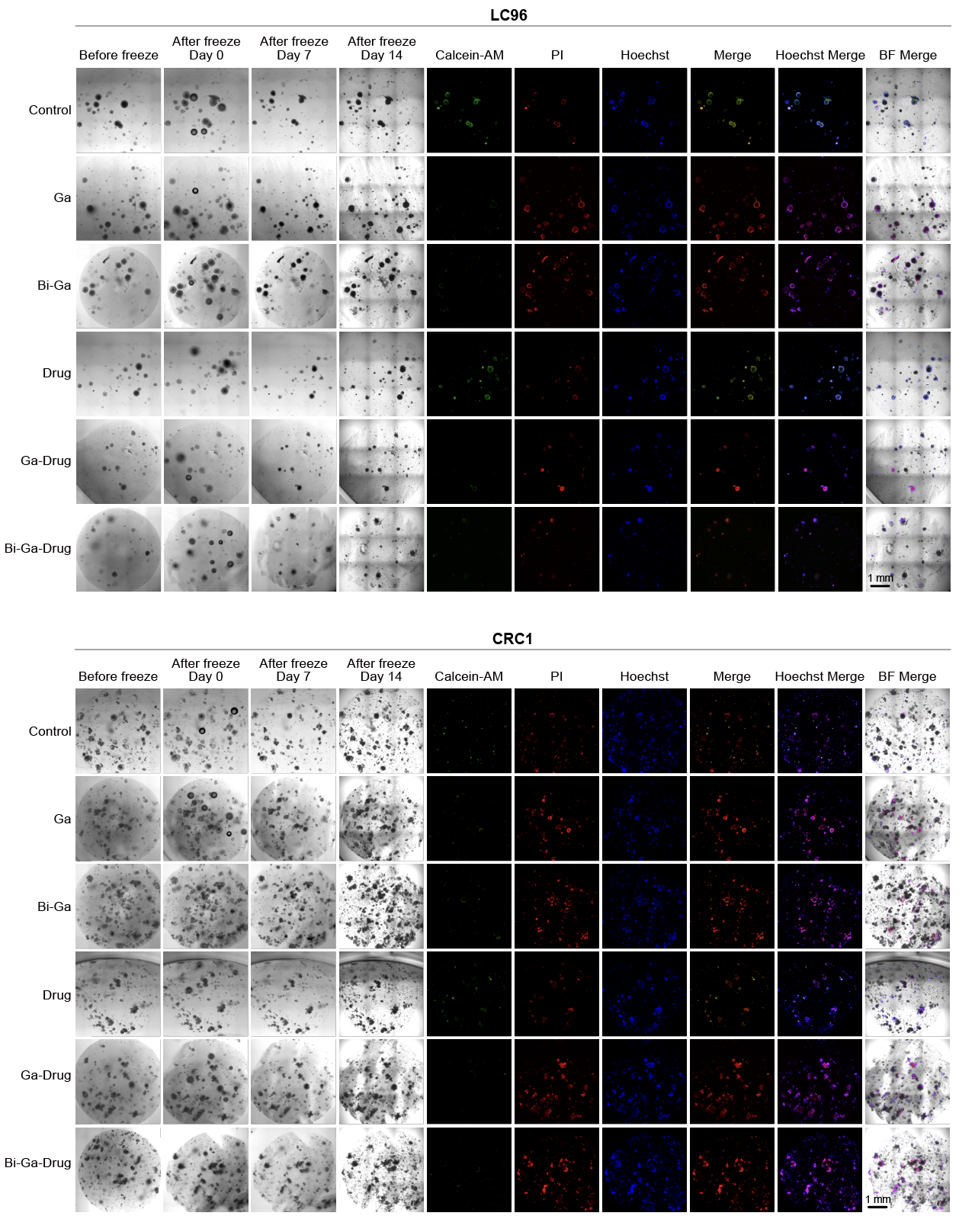


**Figure S16.** Morphological and viability assessment of LC96 and CRC1 organoids following cryo-treatment. Bright-field images of LC96 and CRC1 organoids under 6 conditions at pre-freezing, immediately after freezing, day 7 post-freezing, and day 14 post-freezing, and fluorescence images of live/dead staining at day 14 show live cells labeled with Calcein-AM (green), dead cells with PI (red), and cell nuclei with Hoechst (blue). The 6 groups of conditions including Control, Ga, Bi-Ga, chemotherapeutic drug, Ga combined with chemotherapeutic drug, and Bi-Ga combined with chemotherapeutic drug. Scale bar: 1 mm


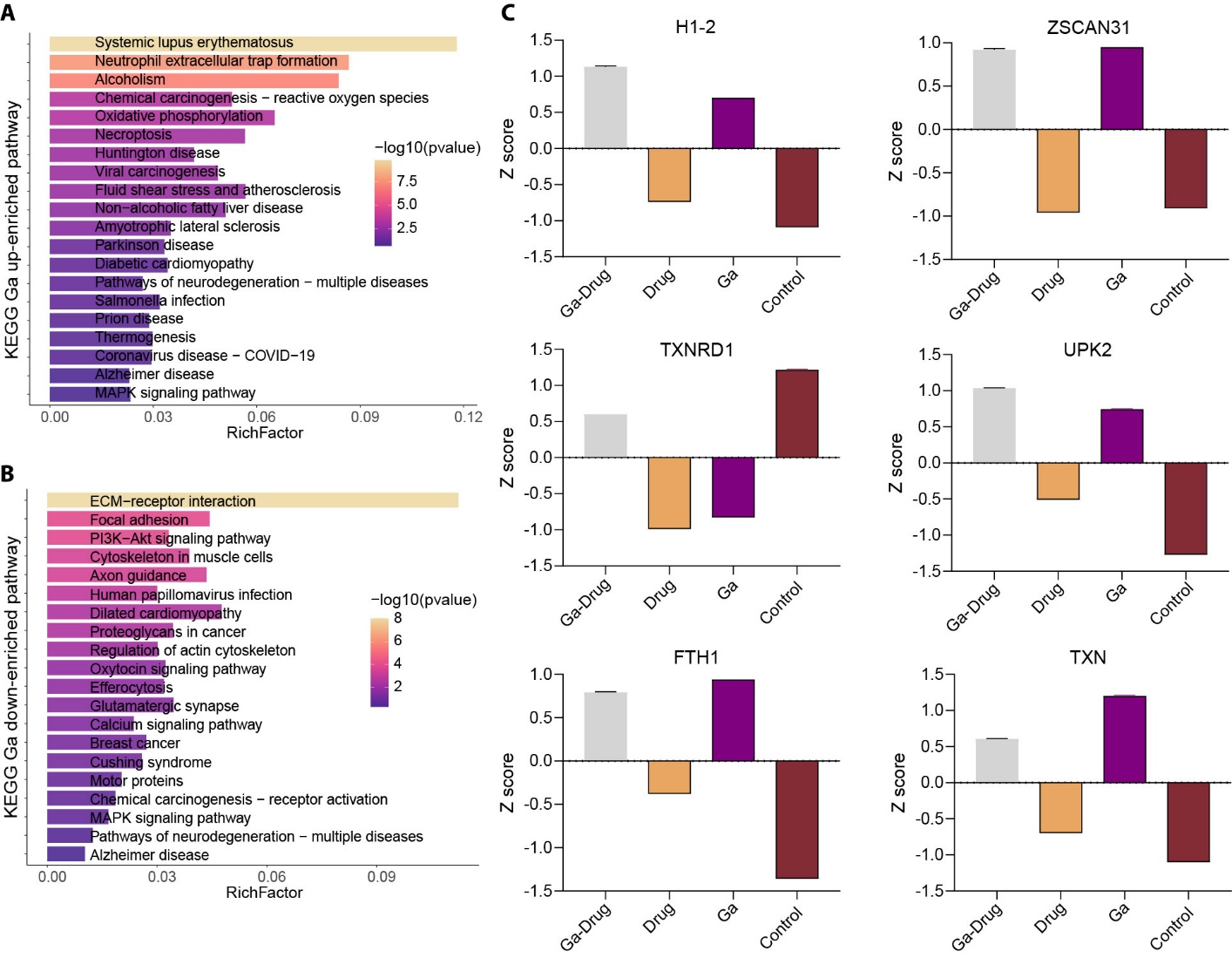


**Figure S17.** Additional anti-tumor effect on CRC organoids by Ga cryo-treatment. (**A**) KEGG up-enriched pathway by Ga cryo-treatment on CRC organoids. (**B**) KEGG down-enriched pathway by Ga cryo-treatment on CRC organoids. (**C**) Z-score analysis of selected DEGs by Ga cryo-treatment on CRC organoids.


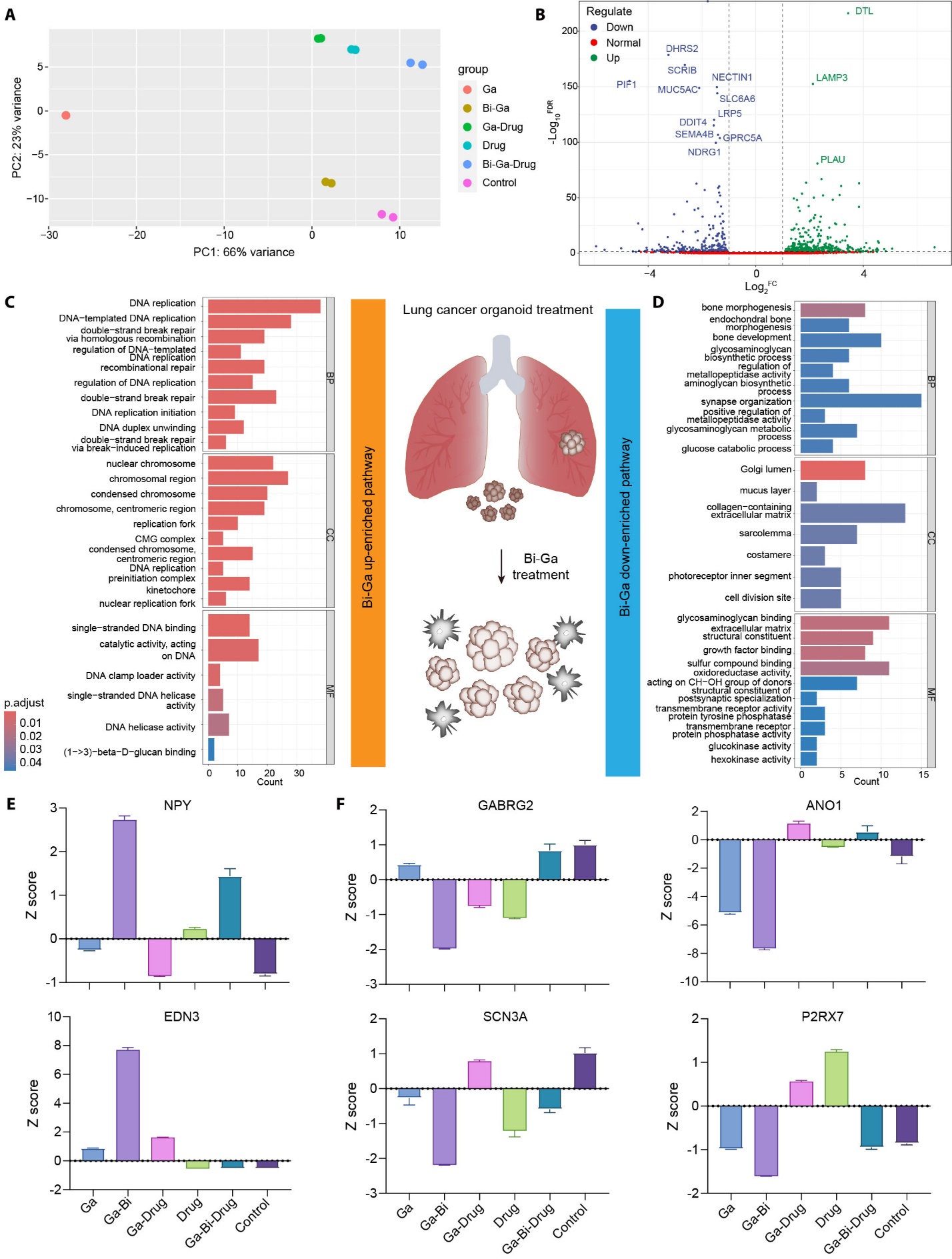


**Figure S18.** Additional anti-tumor effect on LC organoids by Bi-Ga cryo-treatment. (**A**) PCA plot of different cryo-treatment on LC organoids. (**B**) Volcano plot of Bi-Ga cryo-treatment on LC organoids. (**C**) GO up-enriched pathway by Bi-Ga cryo-treatment on LC organoids. (**D**) GO down-enriched pathway by Bi-Ga cryo-treatment on LC organoids. (**E**) Z-score analysis of selected up-regulated DEGs by Bi-Ga cryo-treatment on LC organoids. (**F**) Z-score analysis of selected down-regulated DEGs by Bi-Ga cryo-treatment on LC organoids.


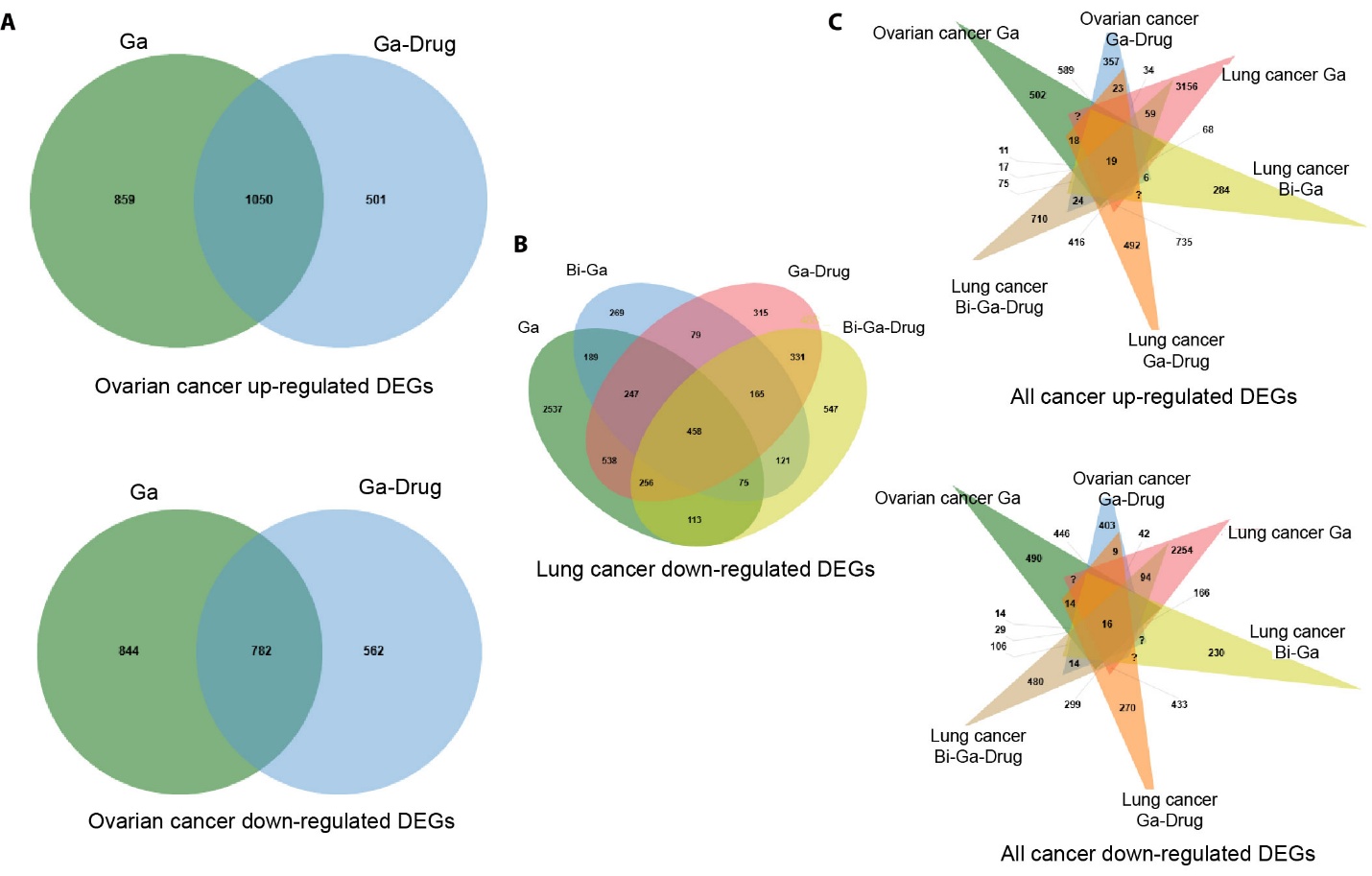


**Figure S19.** Overlap relationship of intervention effect on both CRC and LC organoids by LM cryo-treatment. (**A**) Venn diagram of up and down-regulated DEGs by LM cryo-treatment on CRC organoids. (**B**) Venn diagram of up and down-regulated DEGs by LM cryo-treatment on both CRC and LC organoids. (**C**) Venn diagram of up and down-regulated DEGs by all tumor organoids.


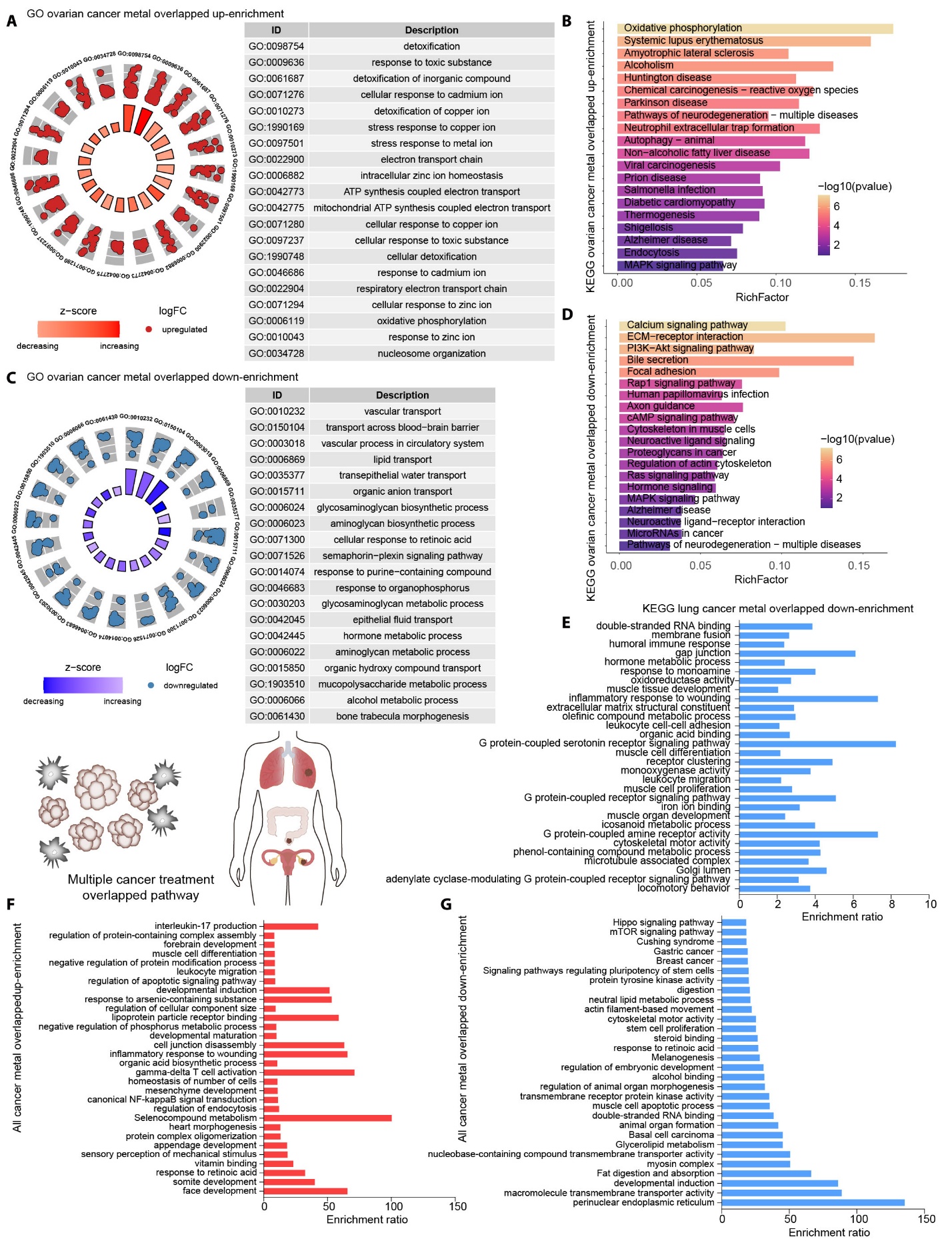


**Figure S20.** Overlap anti-tumor effect on both CRC and LC organoids by LM cryo-treatment. (**A**) GO up-enriched pathway by overlapped LM cryo-treatment on CRC organoids. (**B**) KEGG up-enriched pathway by overlapped LM cryo-treatment on CRC organoids. (**C**) GO down-enriched pathway by overlapped LM cryo-treatment on CRC organoids. (**D**) KEGG down-enriched pathway by overlapped LM cryo-treatment on CRC organoids. (**E**) KEGG down-enriched pathway by overlapped LM cryo-treatment on LC organoids. (**F**) GO and KEGG up-enriched pathway by overlapped LM cryo-treatment on both CRC and LC organoids. (**G**) GO and KEGG down-enriched pathway by overlapped LM cryo-treatment on both CRC and LC organoids.


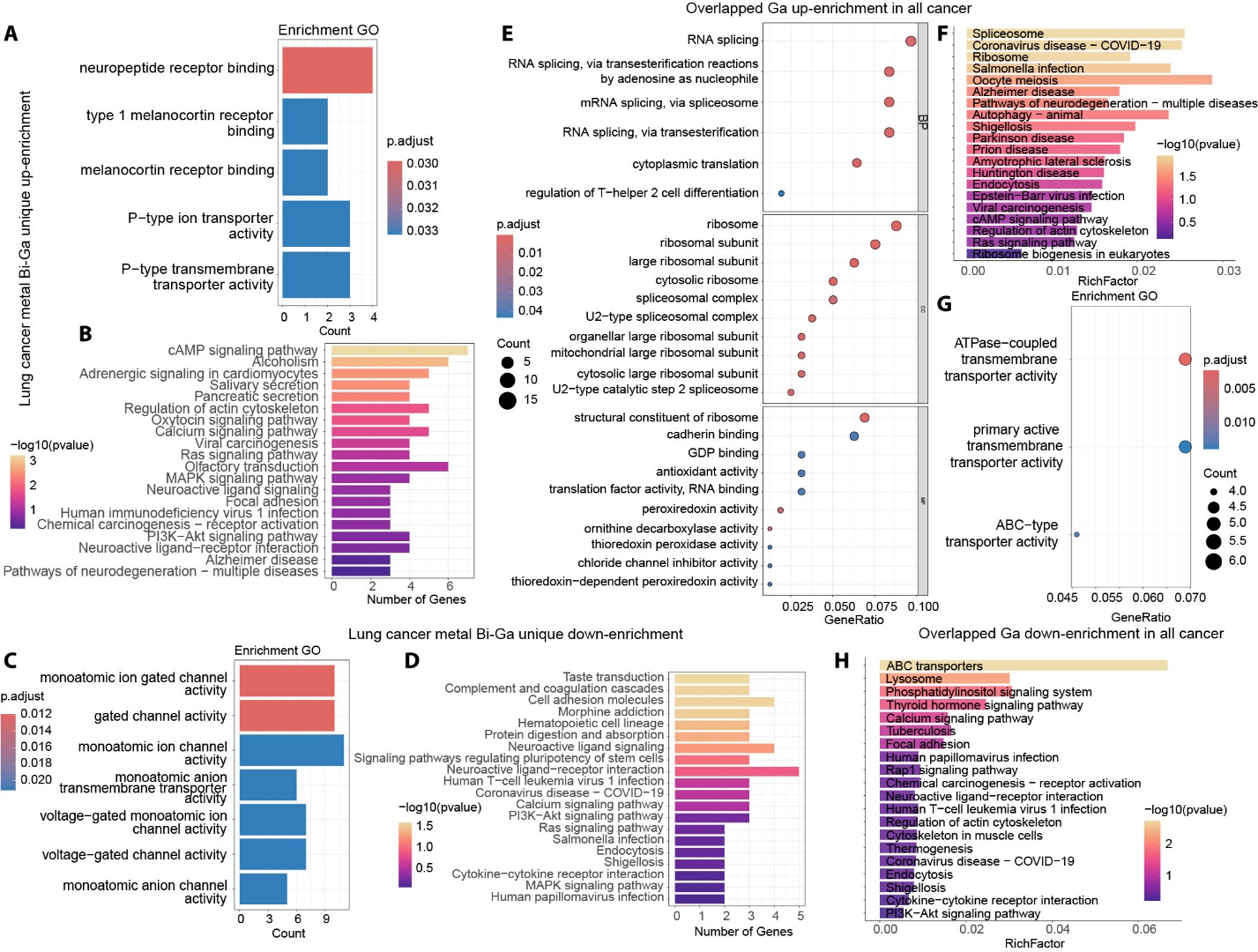


**Figure S21.** Overlap and unique anti-tumor effect on both CRC and LC organoids by Bi-Ga cryo-treatment. (**A**) GO up-enriched pathway by unique Bi-Ga cryo-treatment on LC organoids. (**B**) KEGG up-enriched pathway by unique Bi-Ga cryo-treatment on LC organoids. (**C**) GO down-enriched pathway by unique Bi-Ga cryo-treatment on LC organoids. (**D**) KEGG down-enriched pathway by unique Bi-Ga cryo-treatment on LC organoids. (**E**) Overlapped GO up-enriched pathway by Ga cryo-treatment on both CRC and LC organoids. (**F**) Overlapped KEGG up-enriched pathway by Ga cryo-treatment on both CRC and LC organoids. (**G**) Overlapped GO down-enriched pathway by Ga cryo-treatment on both CRC and LC organoids. (**H**) Overlapped KEGG down-enriched pathway by Ga cryo-treatment on both CRC and LC organoids.


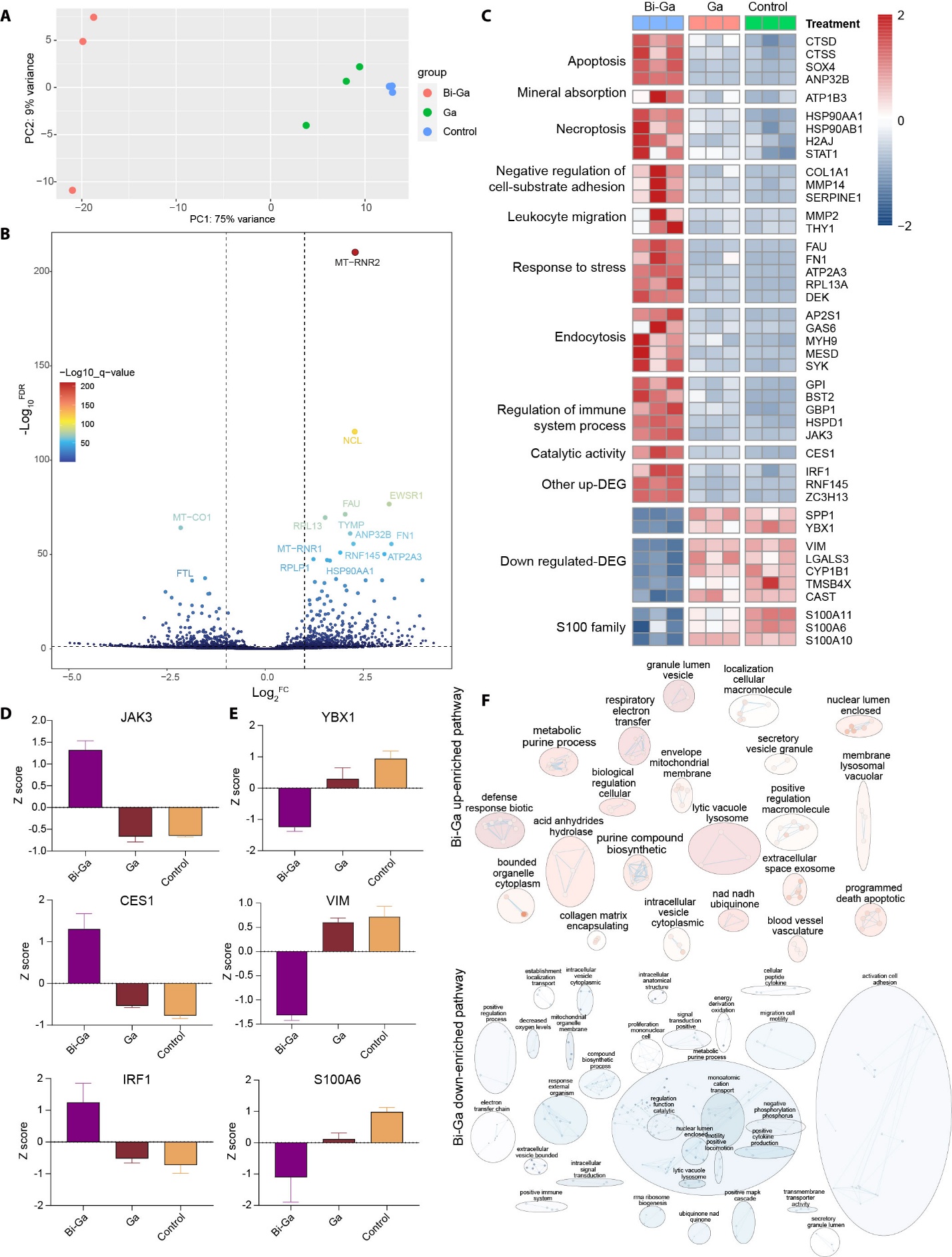


**Figure S22.** Anti-tumor effect on LC organoids with immune compartment by metal cryo-treatment alone. (**A**) PCA plot of different cryo-treatment on LC organoids with immune compartment. (**B**) Volcano plot of Bi-Ga cryo-treatment on LC organoids with immune compartment. (**C**) Heatmap of different cryo-treatment on LC organoids with immune compartment. (**D**) Z-score analysis of selected up-regulated DEGs by Bi-Ga cryo-treatment on LC organoids with immune compartment. (**E**) Z-score analysis of selected down-regulated DEGs by Bi-Ga cryo-treatment on LC organoids with immune compartment. (**F**) GO and KEGG up-enriched pathway by Bi-Ga cryo-treatment on LC organoids with immune micro-environment.


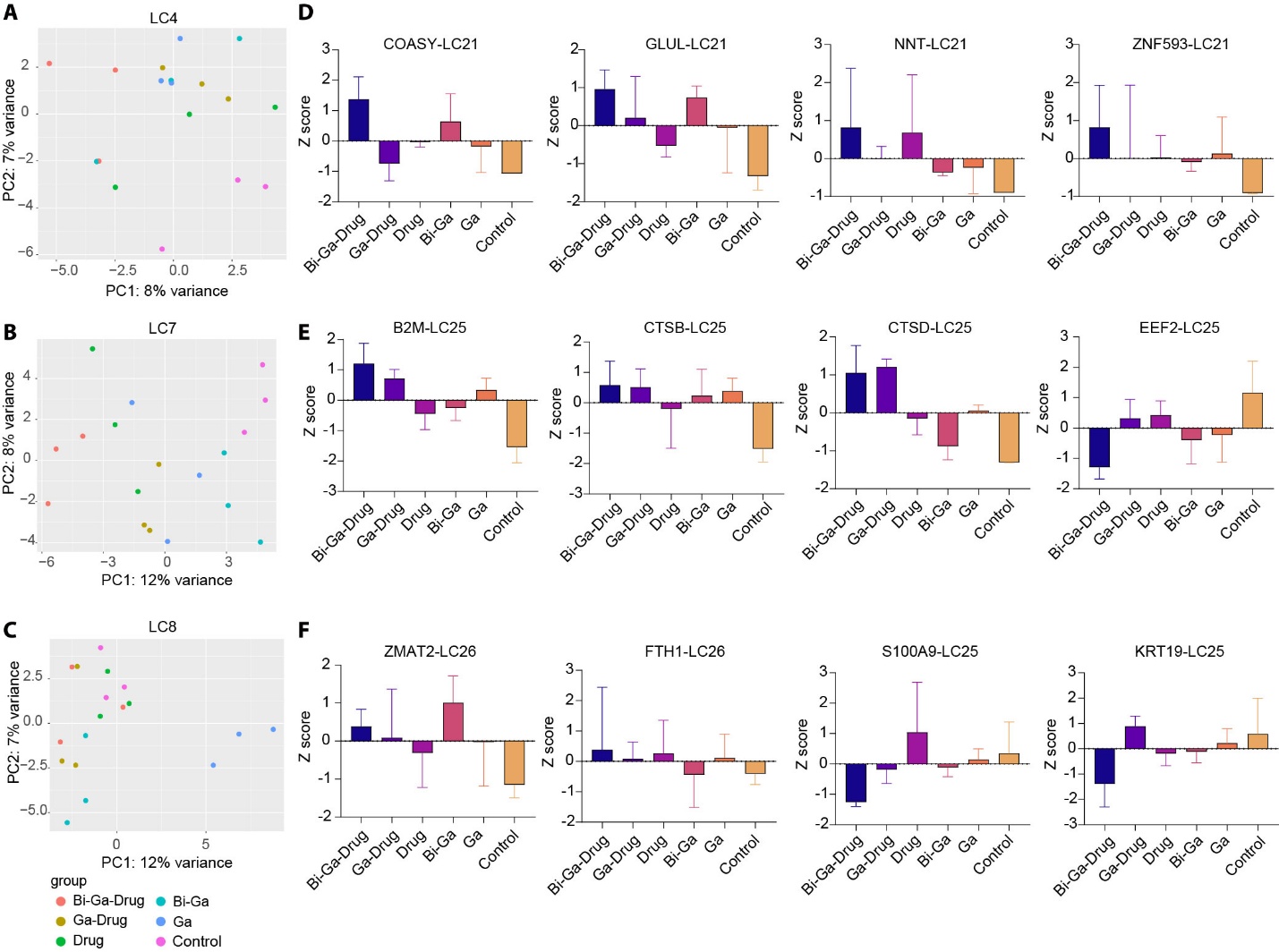


**Figure S23.** Treatment validation of LM cryo-treatment on multiple LC organoids with immune compartment. (**A**) PCA plot of different cryo-treatment on LC4 organoids with immune compartment. (**B**) PCA plot of different cryo-treatment on LC7 organoids with immune compartment. (**C**) PCA plot of different cryo-treatment on LC8 organoids with immune compartment. (**D**) Z-score analysis of selected DEGs by LM cryo-treatment on LC4 organoids with immune compartment. (**E**) Z-score analysis of selected DEGs by LM cryo-treatment on LC7 organoids with immune compartment. (**F**) Z-score analysis of selected DEGs by LM cryo-treatment on LC8 organoids with immune compartment.


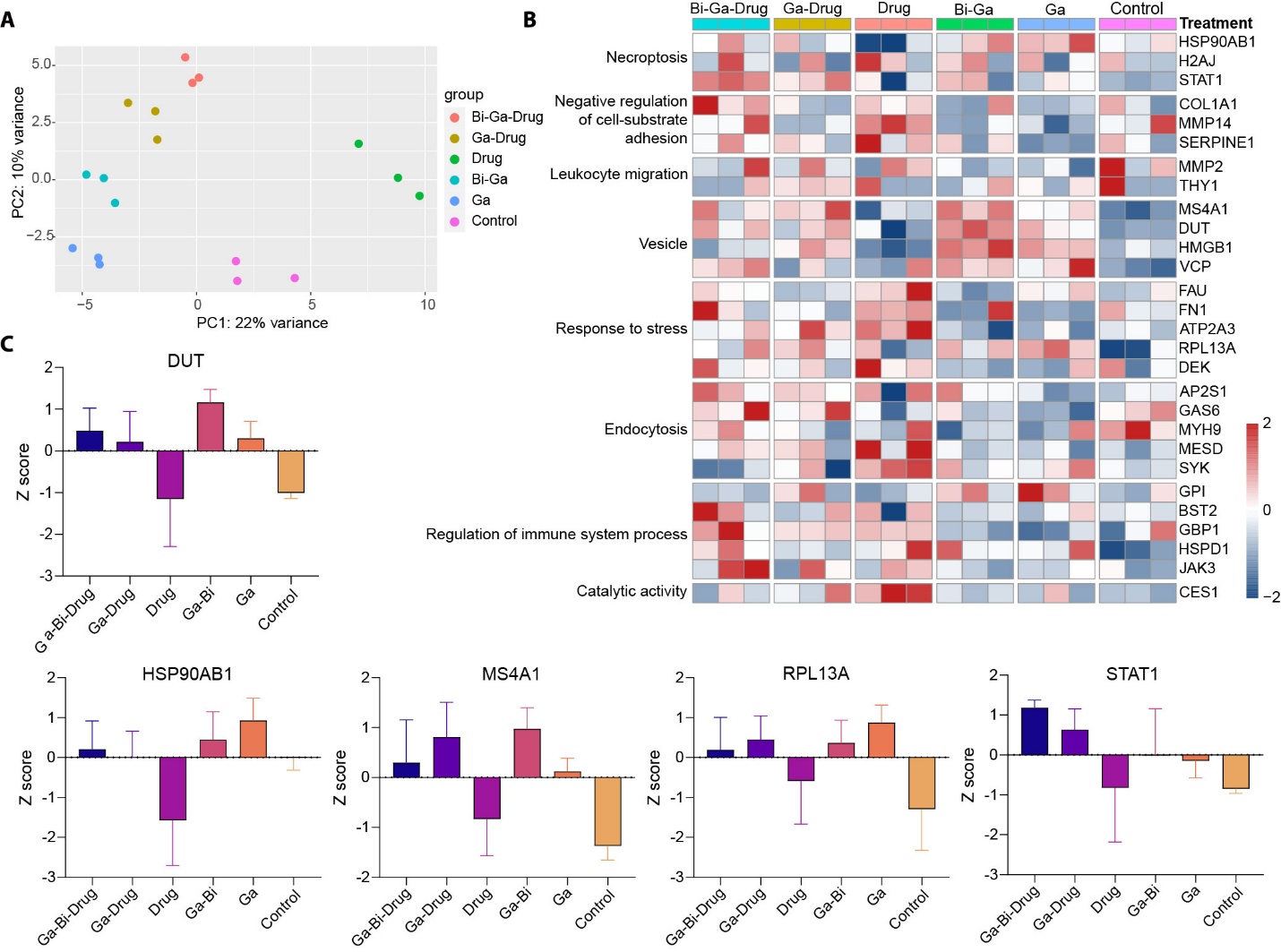


**Figure S24.** Anti-tumor effect on drug resistant LC organoids with immune compartment by LM cryo-treatment. (**A**) PCA plot of different cryo-treatment on drug resistant LC organoids with immune compartment. (**B**) Heatmap of different cryo-treatment on drug resistant LC organoids with immune compartment. (**C**) Z-score analysis of selected DEGs by LM cryo-treatment on drug resistant LC organoids with immune compartment.


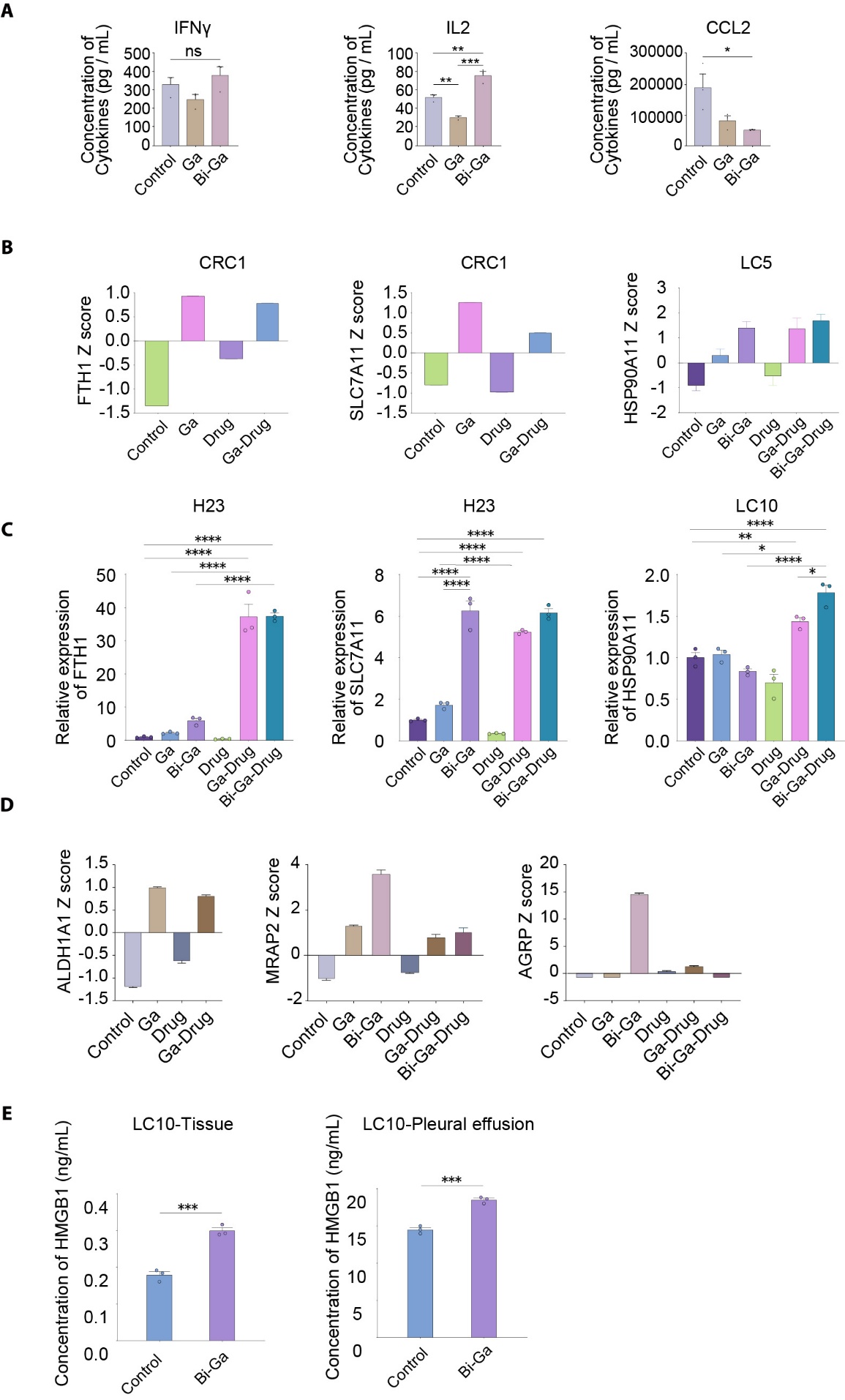


**Figure S25.** (**A**) LEGENDplex immunoassays of immune-activating/suppressive cytokines in the immune microenvironment of LC organoids after Bi-Ga cryo-treatment. (**B**) Z-score analysis of Bi-Ga and Ga cryo-treatment up-regulated DEGs FTH1 and SLC7A11 in CRC organoids and up-regulated DEG HSP90A11 in LC organoids with microenvironment derived from clinical samples. (**C**) mRNA expression levels of FTH1 and SLC7A11 in H23 cells and HSP90A11 of LC10 organoids after cryo-treatment for 10 s. Data are shown as mean ± SEM. Statistical significance was determined by one-way ANOVA. n = 3. (**D**) Z-score analysis of overlapping up-regulated DEGs identified after Ga cryo-treatment in both LC and CRC organoids (ALDH1A1). Z-score analysis of Ga-Bi cryo-treatment unique up-regulated DEGs in LC organoids (MRAP2 and AGRP). (**E**) HMGB1 release from LC10 organoids derived from tissues and Pleural effusion in the supernatant 24 h after cryo-treatment. (n = 3). Data are shown as mean ± SEM. Statistical significance was determined by t test (*P < 0.05, **P < 0.01, ***P < 0.001, ****P < 0.0001; ns, not significant). n = 3.

**
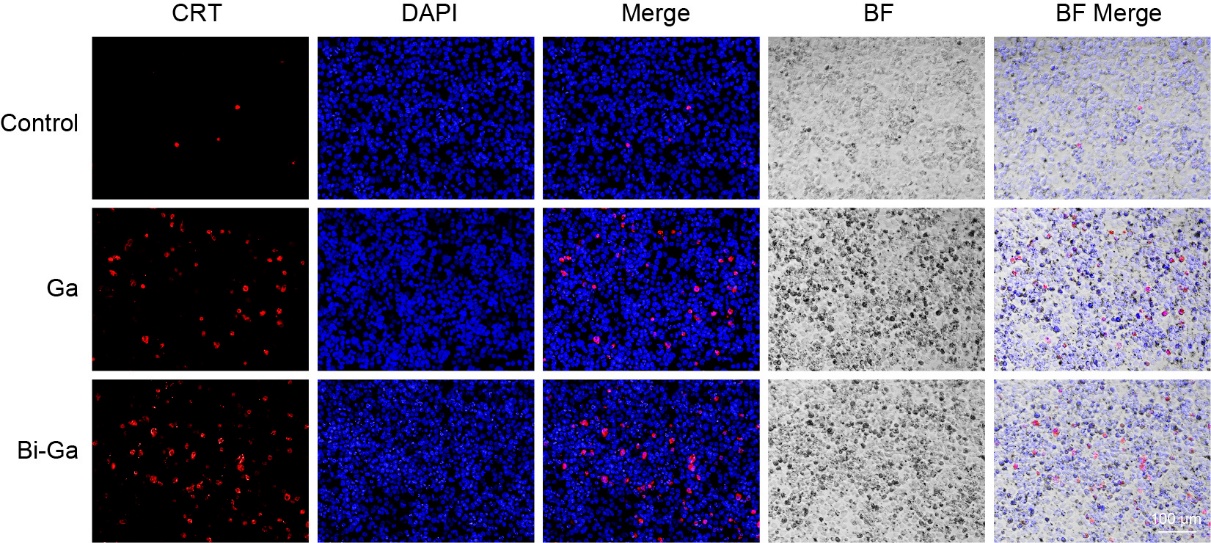
**

**Figure S26.** Immunofluorescence staining showing CRT translocation on H23 cell surface after freezing within 4h. Scale bar 100 μm.


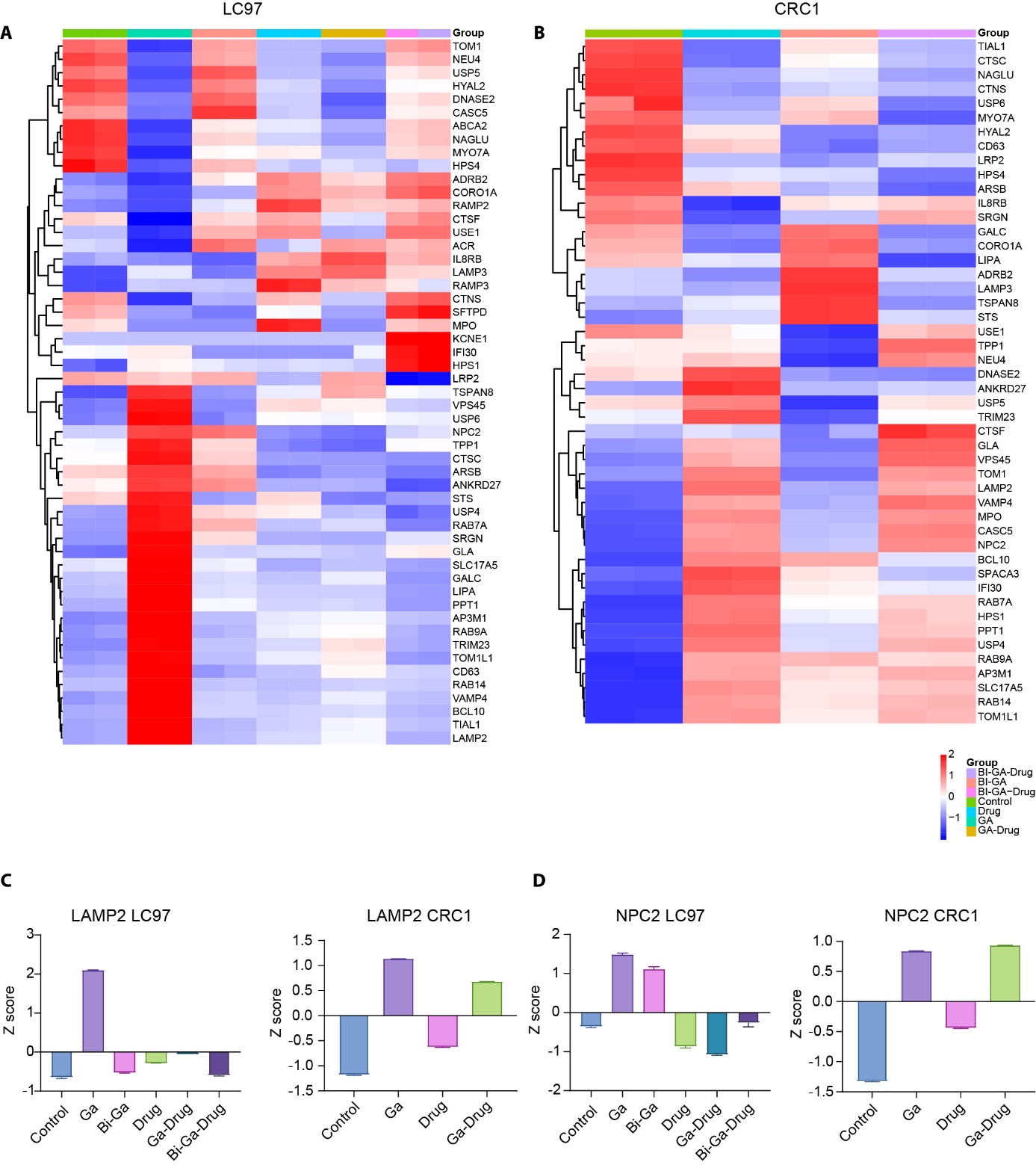


**Figure S27.** (**A**) All lysosomal related gene in LC97 organoids analyzed from bulk RNA sequencing data. (**B**) All lysosomal related gene in CRC1 organoids analyzed from bulk RNA sequencing data. (**C**) Z-score analysis of LAMP2 in LC97 and CRC1 organoids. (**D**) Z-score analysis of NPC2 in LC97 and CRC1 organoids.

**
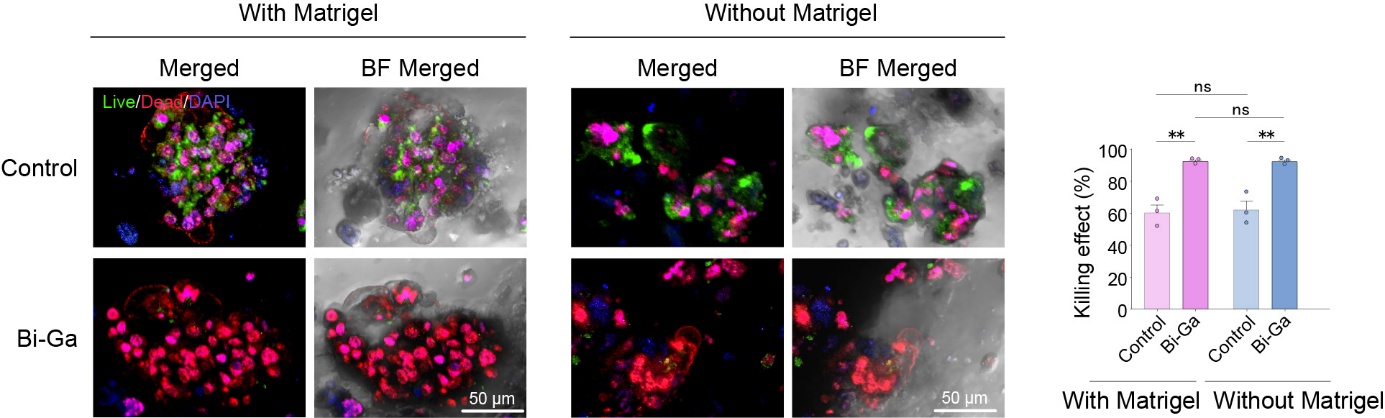
**

**Figure S28.** The live/dead staining of CRC1 organoids in control and Bi-Ga group with and without Matrigel (green: live cells; red: dead cells; blue: cell nucleus) and the killing effect was analyzed by Two way ANOVA to assess the individual and interactive effects of Matrigel and LM on organoids viability. (*P < 0.05, **P < 0.01, ***P < 0.001, ****P < 0.0001; ns, not significant). n = 3.
